# Identification of two rate-limiting steps in the degradation of partially folded immunoglobulin light chains

**DOI:** 10.3389/fcell.2022.924848

**Published:** 2022-08-22

**Authors:** Melissa J. Mann, Ashley R. Flory, Christina Oikonomou, Candace A. Hayes, Chris Melendez-Suchi, Linda M. Hendershot

**Affiliations:** ^1^ St Jude Children’s Research Hospital, Memphis, TN, United States; ^2^ University of Tennessee Health Science Center, Memphis, TN, United States; ^3^ Rhodes College, Memphis, TN, United States

**Keywords:** ER quality control, ERAD, Ig light chain, proteasome, degradation

## Abstract

Antibody monomers are produced from two immunoglobulin heavy chains and two light chains that are folded and assembled in the endoplasmic reticulum This process is assisted and monitored by components of the endoplasmic reticulum quality control machinery; an outcome made more fraught by the unusual genetic machinations employed to produce a seemingly unlimited antibody repertoire. Proper functioning of the adaptive immune system is as dependent on the success of this operation, as it is on the ability to identify and degrade those molecules that fail to reach their native state. In this study, two rate-limiting steps were identified in the degradation of a non-secreted κ light chain. Both focus on the constant domain (C_L_), which has evolved to fold rapidly and very stably to serve as a catalyst for the folding of the heavy chain C_H_1 domain. The first hurdle is the reduction of the disulfide bond in the C_L_ domain, which is required for retrotranslocation to the cytosol. In spite of being reduced, the C_L_ domain retains structure, giving rise to the second rate-limiting step, the unfolding of this domain at the proteasome, which results in a stalled degradation intermediate.

## Introduction

Our immune systems are able to produce antibodies to a seemingly limitless number of antigens. In fact, a recent study estimated that the potential human antibody repertoire may approach a quintrillion unique molecules (Briney et al., 2019). If each antibody was encoded by a separate DNA segment, an absurd number of genome equivalents would be required to produce them. Instead, this incredible feat is made possible through a complex series of molecular manipulations of antibody genes. The variable regions of the heavy and light chain, which provide the antigen recognition capability of antibody, are assembled from three distinct sets of immunoglobulin (Ig) gene families for the heavy chain: Variable (V_H_), Diversity (D_H_), and Joining (J_H_), and two each for the κ and _λ_ light chains (V_κ_, J_κ_ and V_λ_, J_λ_). One DNA segment from each of these heavy and light chain gene families must be successfully recombined, and non-templated nucleotides are added to the ends of the DNA segments prior to their relegation to produce a heavy chain and light chain variable region. In addition, the assembled variable region is subjected to hypermutation to increase antigen affinity (Kenter and Feeney, 2019; Vajda et al., 2021). While this clearly adds to the diversity of the repertoire, from a standpoint of protein folding it represents a veritable nightmare. And yet, absolute fidelity in antibody maturation is required for proper functioning of the immune system.

Like nearly all secreted or cell surface proteins, immunoglobulins are produced in the endoplasmic reticulum, where a dedicated ER quality control (ERQC) system assists and monitors the maturation of nascent proteins. Monomeric IgG antibodies are covalently assembled from two identical γ heavy chains and two identical either κ or λ light chains, which possess four and two Ig domains, respectively, whereas pentameric IgM antibodies are covalently assembled from ten μ heavy chains consisting of five Ig domains, ten light chains, and a J or joining chain. Each Ig domain is approximately 100 amino acids in length and folds into an anti-parallel β barrel structure that is secured with a disulfide bond between highly conserved cysteines in strands 2 and 6 (Oreste et al., 2021). Antibodies have been subjected to numerous *in vitro* and *in vivo* folding studies that have provided significant understanding of the molecular and cellular checkpoints that ensure fidelity of their maturation (Feige et al., 2010). These studies reveal that although most Ig domains can fold and form their intra-domain disulfide bond independently or after homodimerization (Lilie et al., 1994), the first constant domain of the heavy chain (C_H_1) domain is unique in that it remains reduced (Lee et al., 1999) and unstructured (Feige et al., 2009) prior to assembly with a light chain. The unfolded C_H_1 domain reacts with BiP, which serves to retain the incompletely assembled heavy chain in the ER, and deletion of the C_H_1 domain results in the secretion of partially assembled antibody intermediates (Hendershot et al., 1987). Contact with the well-folded constant domain C_L_ of a light chain nucleates oxidative folding of the C_H_1 domain, allowing the completely folded and assembled antibody to be released from BiP and secreted (Feige et al., 2009). In the case of pre-B cells, the surrogate light chain is responsible for associating with the C_H_1 domain of the μ chains, and deletion of the C_H_1 domain of the μ heavy chain locus adversely affects B cell development (Shaffer and Schlissel, 1997). Thus, checkpoints for Ig gene rearrangements are also assessed through the protein quality control system and focus on the ability of a well-folded C_L_ domain to induce folding of the C_H_1 domain.

Given the unusual mechanisms needed to produce the vast antibody repertoire, as well as the extremely high biosynthetic rate achieved in plasma cells, it is to be expected that some genetic manipulations, and even random mistakes during normal synthesis, will produce antibody subunits that fail to fold or assemble properly. The presence of a single unfolded domain requires that the heavy or light chain be targeted for degradation, which occurs through the actions of the ER associated degradation (ERAD) system (Wu and Rapoport, 2018; Needham et al., 2019). Once the decision has been made to degrade an unfolded/misfolded client, it must be targeted and inserted into a protein channel, referred to as the retrotranslocon or dislocon, for extraction to the cytosol where it will be degraded by the proteasome. A number of components of the retrotranslocon have been identified, although it appears there is some heterogeneity in the composition of individual retrotranslocons (Lilley and Ploegh, 2005; Carvalho et al., 2006; Vembar and Brodsky, 2008; Olzmann et al., 2013). One of the translocon components, Hrd1, is a multi-pass integral membrane protein that forms part of the channel itself (Carvalho et al., 2010; Stein et al., 2014). Hrd1 possesses E3 ubiquitin ligase activity with the RING domain oriented to the cytosol (Gardner et al., 2000; Deak and Wolf, 2001). Upon emerging into the cytosol, the ERAD client becomes poly-ubiquitinated, which can occur on a number of amino acids, including serine, threonine, and cysteine, in addition to the prototypical lysine residue (Cadwell and Coscoy, 2005; Wang et al., 2007; Ishikura et al., 2010; Shimizu et al., 2010). In addition to Hrd1, a limited number of other ER-associated E3 ligases have been identified (Kostova et al., 2007). The attached ubiquitin chain provides a recognition motif for the p97/VCP complex (Meyer et al., 2002) that is associated with the ER membrane (Ye et al., 2004; Ye et al., 2005). The AAA-ATPase, p97, provides the energy to extract ERAD clients from the ER membranes. With the possible exception of the cholera toxin A1 subunit (Kothe et al., 2005; Moore et al., 2013), all integral membrane, as well as soluble, luminal ERAD clients examined thus far require the activity of p97 for their disposal.

To understand the cellular checkpoints and requirements for the degradation of an Ig subunit that fails to mature properly and how a well-folded domain affects these, we chose the NS1 Ig light chain. It is comprised of a variable region (V_L_) that forms its disulfide bond but folds unstably and a well-folded, oxidized constant domain (C_L_). We found that although the V_L_ domain must be reduced (Knittler et al., 1995; Okuda-Shimizu and Hendershot, 2007) and is the target of ubiquitination in the cytosol (Shimizu et al., 2010), reduction of the C_L_ domain represents a more significant rate-limiting step in its retrotranslocation. The C_L_ domain either maintains or re-achieves structure once in the cytosol explaining the lack of ubiquitination of this domain. Unfolding of the C_L_ at proteasome represents a second rate-limiting step in its degradation.

## Materials and methods

### DNA constructs and generation of mutants

The non-secreted murine NS1 κ LC (Skowronek et al., 1998) and the ubiquitination-deficient NS1-V_L_STK- (Shimizu et al., 2010) were cloned in pSVL vector. Single N-linked glycosylation consensus sites (N-X-S/T) were engineered throughout NS1 in order to monitor deglycosylation, which occurs after substrate retrotranslocation to the cytosol. Generation of these mutants was performed using the PWO enzyme (11644955001, Roche) or the Q5 mutagenesis kit (E0554S, NEB, Ipswich, MA). When glycosylation sites were added to the folded C_L_ domain, the mutations were introduced on β turns or loops, which were mapped using the available crystal structure for the mouse C_κ_ domain (UniProtKB - P01837) to minimize adverse effects on the natural folding of this domain. A cytosolically expressed NS1 (ΔssNS1) was engineered by removing the ER targeting signal sequence, and replacing methionines present at positions 4, 11, 13 with alanines to prevent possible alternative translation initiation sites. Primers used to create the amino acid changes in these constructs are found in Table 1. The NS1-Vmut and NS1-Cmut constructs were created in the pSVL vector using PCR-mediated, site-directed mutagenesis to replace the two cysteine codons in either the V_L_ or C_L_ domains with serine codons. The PDIA6 construct in the pCDNA3 vector was generously provided by Dr. Neil Bulleid (University of Glasgow, Scotland, United Kingdom), and ERdj5, also in pCDNA3, was a kind gift from Dr. Kazuhiro Nagata (Kyoto Sangyo University, Japan). Wild type p97 and the ATP hydrolysis-defective mutant p97QQ, each in the pcDNA3 vector, were considerate gifts from Dr. Yihong Ye (NIDDK, United States). The Hrd1 mutant deficient in ubiquitin ligase activity (Hrd1 C291S in pcDNA3) was kindly supplied by Dr. Yuval Reiss (Proteologics, Israel).

**TABLE 1:** Primers used to make NS1 mutants.

Site/AA substitution	Forward primer	Reverse primer
NS1-N28/V30T	GCC​AGT​GAG​AAT​GTG​acc​ACT​TAT​GTT​TCC​TGG	CCA​GGA​AAC​ATA​AGT​ggt​CAC​ATT​CTC​ACT​GGC
NS1-N53/Y55T	GCA​TCC​AAC​CGG​acc​ACT​GGG​GTC​CCC	GGG​GAC​CCC​AGT​ggt​CCG​GTT​GGA​TGC
NS1-N100/G100N	CAC​GTT​CGG​Aaa​cGG​GAC​CAA​GC	TACGGATAGCTGTAACCC
NS1-N129/G129N	AAC​ATC​TGG​Aaa​tGC​CTC​AGT​CGT​GTG	AACTGCTCACTGGATGGT
NS1-N157/V159T	ACA​AAA​TGG​Cac​cCT​GAA​CAG​TTG	CGTTCACTGCCATCAATC
NS1-Vmut and Cmut-N157/V159T	ACA​AAA​TGG​Cac​tCT​GAA​CAG​TTG​GAC	CGTTCACTGCCATCAATC
NS1-N170/D170N	GGA​CAG​CAA​Aaa​tAG​CAC​CTA​CA	TGA​TCA​GTC​CAA​CTG​TTC​AG
NS1-VLSTK- N28/V30T	TGA​GAA​TGT​Gac​cGC​TTA​TGT​TGC​CTG​GTA​TCA​ACA​GAG​ACC​AGA​G	GCGGCCCTGCAGGCCAAG
NS1-VLSTK- N100/G100N & A102T	gac​cAG​GCT​GGA​AAT​AAG​ACG​GG	cca​ttT​CCG​AAC​GCG​TAC​GGA​TA
ΔssNS1(removing ER target SS)	ACC​GGA​TCG​ATC​CCT​CGA​CCT​GCA​GAT​GGG​GAA​CAT​TGT​AAT​GAC​CCA​ATC​TCC​CA	TGG​GAG​ATT​GGG​TCA​TTA​CAA​TGT​TCC​CCA​TCT​GCA​GGT​CGA​GGG​ATC​GAT​CCG​GT
ΔssNS1 (M4A,M11A,M13A)	aaa​tcc​gct​tcc​gct​TCA​GTA​GGA​GAG​AGG​GTC	ggg​aga​ttg​ggt​agc​TAC​AAT​GTT​CCC​CAT​CTG

### Cell culture and transfections

293T human embryonic kidney cells were grown in Dulbecco’s modified Eagle’s medium (DMEM; 15–013-CV, Corning–cellgro, Manassas, VA) supplemented with 10% (v/v) heat-inactivated fetal bovine serum (FBS; S11150, Atlanta biologicals, Flowery Branch, GA), 2 mM L-glutamine (25–005-CI, Corning), and a 1% (v/v) antibiotic-antimycotic solution (25 μg/ml amphotenicin B, 10,000 μg/ml streptomycin, and 10,000 units of penicillin; Cellgro/Mediatech, Manassas, VA) at 37°C and 5% CO_2_. 293T cells were plated 24 h prior to transfection, which was performed using GeneCellin (GC5000, BioCellChallenge, Toulon, France) according to the manufacturer’s protocol. For all analysis, 1 μg of each indicated ERAD substrate was used per p60 dish. When p97WT, p97QQ or Hrd1C291S was co-expressed, 1.5 μg of each plasmid was used and an equal amount of empty pcDNA3.1 vector was used in the control samples. When PDIA6 or ERdj5 was co-expressed, 2 μg of the plasmid was used. The P3U.1 murine plasmacytoma cells, which naturally synthesize the NS1 κ LC, were grown in complete DMEM supplemented with 55 µM 2-mercaptoethanol (21985023, Gibco, Grand Island, NY) at 37°C and 5% CO_2_. The Ag8.653 chain-loss variant cell line was grown in the same conditions and was used as a negative control for experiments with P3U.1 cells.

### Metabolic labeling and immunoprecipitation experiments

Twenty-four hours after transfection, cells were pre-incubated at 37°C for 30 min in 2 ml media consisting of 1x DMEM without Cys and Met (Cys^−^/Met^−^) (17-204CI, Corning), which was supplemented with 1% L-glutamine and 10% FBS dialyzed against PBS to deplete amino acids. Cells were labeled with Express [^35^S] Labeling Mix (NEG072-007, PerkinElmer) for 15 min. After labeling, culture dishes were placed on ice, washed once with 2 ml cold DPBS (21–031-CV, Corning), and cells were re-incubated in 2 ml of chase media (complete DMEM supplemented with 2 mM each of unlabeled methionine (M5308, Sigma) and cysteine (C7352, Sigma). For the NS1-N157 deglycosylation experiments, cells were pre-incubated in 10 µm MG132 for 2 h and 15 min before starving. The starving, labeling and chase media all contained 10 µm MG132. Zero time points were lysed immediately in Nonidet P-40 lysis buffer (NP-40: 50 mM Tris/HCl pH 7.5, 150 mM NaCl, 0.5% Nonidet P40, 0.5% sodium deoxycholate, 0.1 mM PMSF, 1X complete protease inhibitor tablets without EDTA), and all other plates were returned to 37°C for the indicated time points. Lysates were clarified by centrifugation at 18,000 g and incubated with goat anti-mouse κ (SouthernBiotechonology) overnight at 4°C on a rotator. Protein A agarose beads (CA-PRI-0100, Repligen) were added for an hour the following morning. After washing the beads 3 times with Nonidet P-40 wash buffer (NP-40 lysis buffer with 400 mM NaCl), proteins were eluted with 2x sample buffer at 95°C. 30% of immunoprecipitated proteins were electrophoresed on polyacrylamide gels and transferred to PVDF membranes. Dry membranes were exposed to BAS storage phosphor screens (Cytiva, 28956475) and scanned using a phosphor imager (Typhoon FLA 9500 GE Healthcare) to detect signals. Signals were quantified with the ImageQuant TL software (Cytiva). Non-transfected cells were used to subtract any background signal from the samples.

### Western blot analysis

Clarified NP40-solubilized lysates were mixed with reducing sample buffer (1:1), and samples were separated on 13% SDS-polyacrylamide gels for nearly all analyses. Exceptions include studies to determine the oxidation status of our substrates in which 15% gels were used, and the Proteinase K digestion and pulse-chase experiments where 12% gels were used. Post-electrophoresis, proteins were transferred to PVDF membranes (IPFL00010, Millipore). Membranes were fixed with methanol, blocked with gelatin wash buffer, and incubated overnight with the indicated primary immune reagents in blocking buffer followed by species-specific secondary reagents, also in blocking buffer. Western blots with HRP-conjugated secondary antibodies were developed using the Pierce™ ECL (32106, Thermo Fisher Scientific, Waltham, MA), and for quantitative analysis they were scanned with the LI-COR Fc Odyssey scanner (LI-COR, Lincoln, NE). When secondary antibodies conjugated to IRDye680 or IRDye800 (Li-COR) were employed, protein bands were detected using the LI-COR Odyssey CLx Imager (LI-COR Biosciences). Analysis and quantification were performed with the Image Studio Lite software. For experiments where the oxidation status of clients was studied, cells were lysed in complete NP-40 buffer additionally supplemented with 20 mM NEthylmaleimide (NEM; E3876-5G, Sigma-Aldrich, St. Louis, MO).

For co-immunoprecipitation-coupled western blot experiments, 3 × 10^6^ P3U.1 (κ LC expressing) or Ag8.653 (Ig^−^) cells were used. To detect association of proteins, cells were incubated with a membrane-permeable cross-linking agent prior to lysing as described previously (Meunier et al., 2002). Briefly, cells were washed and resuspended in cold Hepes buffer (25 mM Hepes-KOH, pH 8.3 and 125 mM KCl). A 5 mg/ml solution of 3,3′-Dithiodipropionic acid di (N-hydroxysuccinimide ester) (DSP) (Sigma-Aldrich), was freshly prepared in DMSO and added to the cells to achieve a final concentration of 150 μg/ml. Cells were incubated on ice for 1h with occasional shaking. After quenching with 100 mM glycine final concentration, cells were lysed in RIPA buffer (10 mM Tris/HCl pH 7.5, 150 mM NaCl, 1% Nonidet P40, 0.2% sodium deoxycholate, 0.1% SDS, 0.1 mM PMSF, 1X complete protease inhibitor tablets without EDTA). LCs were immunoprecipitated overnight by incubating with anti-κλ-conjugated agarose beads (custom made by SouthernBiotech using their antibodies 1050–01 and 1060–01). LCs and associated proteins were eluted from the beads with 2x sample buffer, electrophoresed, and interacting proteins were analyzed by western blotting.

### Antibodies

The following antibodies were used for blotting: polyclonal goat anti-mouse κ LC (1050–01, SouthernBiotech); mouse anti-GAPDH (MAB374, Millipore); mouse anti-Hsc70 (B-6) (sc-7298, Santa Cruz Biotechnology), mouse anti-VCP/p97 (ab11433, abcam, Cambridge, MA), mouse anti-20S proteasome subunit alpha (C8) (PW8110, Biomol, Hamburg, Germany), rabbit anti-PSMC2 (HPA019238, Sigma-Aldrich), mouse anti-actin (A5441, Sigma-Aldrich), rabbit anti-PDIA6 (NBP157999, Novus Biologicals). HRP-conjugated antibodies were used at 1:10,000 dilution and include goat anti-rabbit (sc-2054), donkey anti-goat (sc-2020), and goat anti-mouse (sc-2031) all purchased from Santa Cruz Biotechnology. The rabbit anti-ERdj3 antibody was produced in our lab (Shen and Hendershot, 2005). IRdye^®^ secondary antibodies (LI-COR Biosciences, all 1:20,000): goat anti-mouse IgG (925–32210), goat anti-rabbit IgG (925–68071), and donkey anti-goat IgG (926–68074).

### Cycloheximide chase and deglycosylation experiments

For cycloheximide (CHX) and/or MG132 chase experiments, cells were treated with 10 μΜ ΜG132 or 100 μg/ml CHX for the indicated times. The cells were lysed in 0.5 ml of NP40 lysis buffer, clarified by centrifugation at 18,000 × g for 15 min, and 1.5% of the resulting lysate was analyzed by western blotting. To remove N-linked glycans from the NS1 glyco-mutant constructs, we treated samples with Endo H (P0702L, NEB) according to the manufacturer’s protocol. Samples were mixed with 2x Laemmli sample buffer and analyzed by western blotting.

### Assessment of disulfide bond content

To monitor the oxidation status of the various proteins, cells expressing the constructs of interest were washed twice with PBS containing 20 mM NEM (E3876-5G, Sigma-Aldrich) and lysed in 0.5 ml NP-40 supplemented with 0.1 mM PMSF, 1X complete protease inhibitor tablets without EDTA and 20 mM NEM.

### Concanavalin A binding experiments

Concanavalin A (Con A) beads (AL-1003, VECTOR LABORATORIES, Burlingame, CA) were used to separate glycosylated and non-glycosylated species. 293T cells were grown in p60 dishes and transfected with the mono-glycosylated NS1 client (NS1-N100). After 24 h, the cells were incubated with 10 μM MG132 in DMSO or in an equal volume of DMSO alone as a control for 3.5 h and were then lysed in 0.5 ml of NP-40 lysis buffer supplemented with 1 mM CaCl_2_, 1 mM MgCl_2_, 1 mM MnCl_2_, 0.1 mM PMSF and 20 mM NEM. Clarified lysates were incubated overnight at 4°C with 2:1 v/v Con A bead slurry. Beads were centrifuged (500 × g for 5 min) and the supernatant (unbound) was collected and mixed with an equal volume of 2x non-reducing Laemmli buffer for analysis of the non-glycosylated proteins. The glycosylated proteins (bound) were eluted from the beads with a volume of 1x non-reducing Laemmli buffer equal to the total volume of the non-bound sample. The resulting pools were analyzed by western blotting.

### Partial proteolysis

Stability of indicated proteins was assessed by partial proteolysis experiments. Cells expressing the indicated constructs were lysed in 0.5 ml NP-40 devoid of protease inhibitors and PMSF. Proteinase K (V302B, PROMEGA, Madison, WI) was added to a final concentration of 20 μg/ml. Digestion was performed on ice for 25 min and was followed by a 5 min incubation with PMSF (5 mM final concentration) (Sigma) to inactivate the Proteinase K. An equal volume of 2x Laemmli sample buffer was added to samples, which were heated to 95°C for 5 min and immediately loaded onto SDS-PAGE gels for western blotting. To determine the glycosylation status of the Proteinase K resistant fragments, the samples were treated with Endo H. At the end of this reaction 2x Laemmli buffer was added, and samples were immediately analyzed by western blotting.

### Mass spectrophotometer analysis

P3U.1 and Ag8.653 cells (1.5 × 10^6^ of each) were incubated with 10 μM MG132 or DMSO for 3.5 h and lysed in NP40 lysis buffer. LCs were isolated by immunoprecipitated overnight with anti-mouse-κλ-conjugated agarose beads (custom made by SouthernBiotech using their antibodies 1050–01, and 1060–01) and washed 3 times in NP40 lysis buffer made to 400 mM NaCl. Beads were resuspended in 20 μL of 2x Lammeli buffer with 2-β mercaptoethanol and heated to 95°C for 5 min. Solubilized proteins were loaded on a precast 4–15% gradient gel (BioRad) and run 1 cm into the gel. The gel was then incubated for 1 h in GelCode-Blue (Thermo Scientific), destained overnight in ultrapure H_2_O at room temperature, and transferred to our Center for Proteomics and Metabolomics core for proteome profiling by spectral counting.

The stained portion of the gel was cut into small pieces and reduced with dithiothreitol to ensure complete breakage of disulfide bonds. Cysteine residues were alkylated by iodoacetamide to allow the recovery of Cys-containing peptides. The gel segment was washed, dried in a speed vacuum, and rehydrated with a buffer containing trypsin and incubated overnight. The next day the digested samples were acidified, and the peptides were extracted multiple times. The extracts were pooled, dried, and reconstituted in a small volume. The peptide samples were loaded on a nanoscale capillary reverse phase C18 column by a HPLC system (Thermo EasynLC 1000) and eluted by a gradient (∼60 min). The eluted peptides were ionized by electrospray ionization and detected by an inline mass spectrometer (Thermo Elite). The MS spectra were collected first, and the 20 most abundant ions are sequentially isolated for MS/MS analysis. This process is cycled over the entire liquid chromatography gradient. Database searches were performed using the Sequest search engine in our in-house SPIDERS software package. All matched MS/MS spectra were filtered by mass accuracy and matching scores to reduce protein false discovery rate to ∼1%. Finally, all proteins identified in one gel lane were combined.

## Results

### The well-folded kappa C_L_ domain is completely retrotranslocated across the ER membrane after proteasome inhibition

**FIGURE 1:**
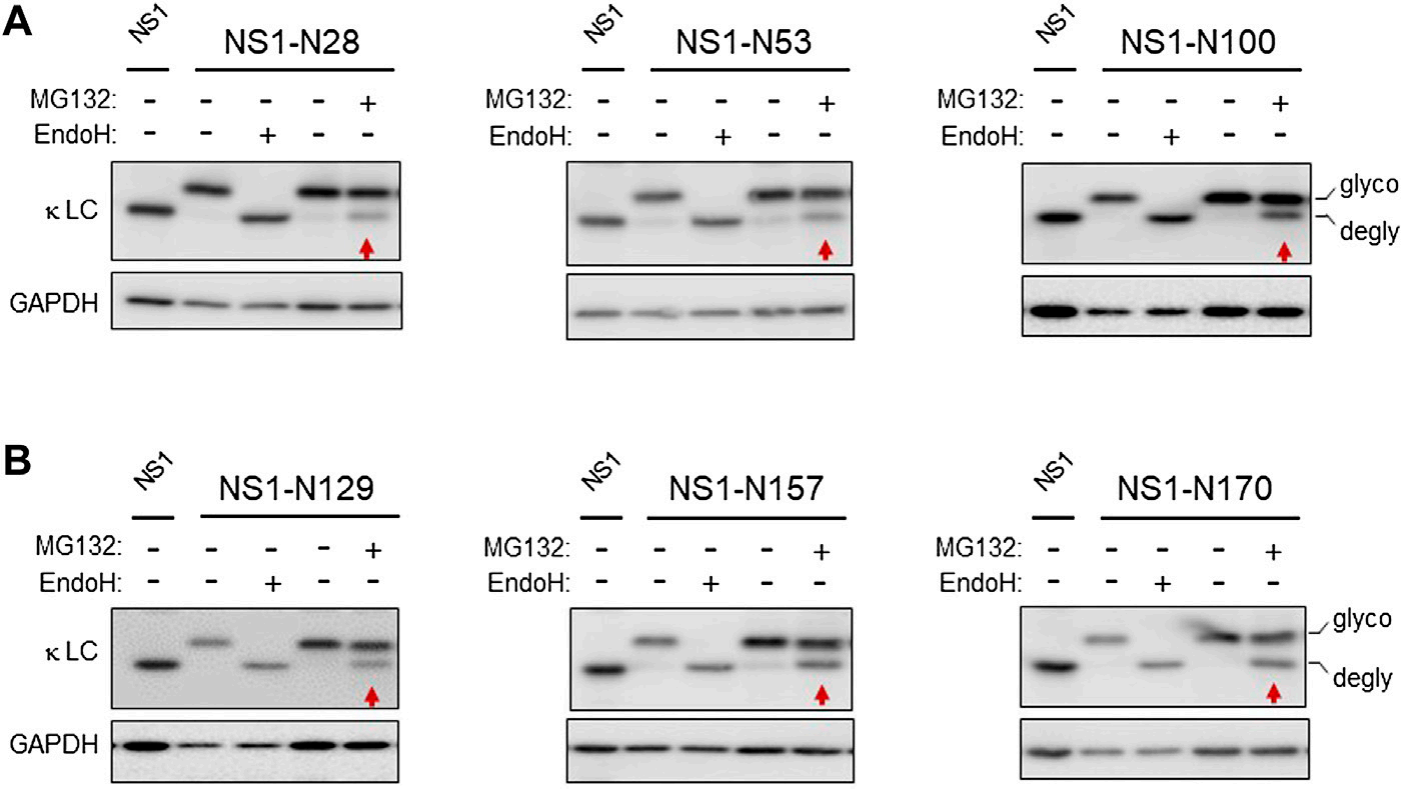
The well-folded domain in NS1 does not impede retrotranslocation. **(A)** Cells were transfected with the indicated NS1 constructs bearing a single N-linked glycan on the V_L_ domain, and 24 h later cell lysates were prepared. Lysates were treated with (+) or without (-) Endo H to identify mobility of the deglycosylated and non-glycosylated forms of these constructs. In each case, the parental, non-glycosylated NS1 was used as a control for the mobility of the non-glycosylated form of NS1. One dish each of cells expressing the indicated glycosylated constructs were incubated with 10 μM MG132 (+) or DMSO control (-) for 3.5 h prior to lysis. Lysates were prepared, and one sample was digested with Endo H (+) as a control for mobility of the unglycosylated protein, and then samples were analyzed by SDS-PAGE-coupled western blotting with a goat anti-mouse κ antisera. Red arrows point at the deglycosylated (DG) species in each panel. **(B)** Cells were transfected with the indicated NS1 glycosylated constructs bearing a single glycan on the C_L_ domain alone and processed as in **(A)**. Non-glycosylated NS1 was used as a control for the mobility of the non-glycosylated form of NS1. In all cases, GAPDH was used as a loading control.

The non-glycosylated NS1 κ light chain has been the focus of multiple ERAD studies (Knittler et al., 1995; Skowronek et al., 1998; Shimizu et al., 2010). Unlike most Ig light chains, it is not secreted in the absence of its partner heavy chain, due to a V_L_ domain that does not fold stably. However, its C_L_ domain folds well, forms its intrachain disulfide bond, and can stimulate the folding of the C_H_1 domain of a γ heavy chain leading to the secretion of a properly antibody (Lee et al., 1999; Vanhove et al., 2001). To determine if the C_L_ domain impeded transport across the ER membrane for degradation, we made use of the fact that N-glycanase 1 is present on the cytosolic side of the retrotranslocon and serves to deglycosylate ERAD clients as they emerge from the retrotranslocon (Suzuki et al., 2016). We engineered a single glycan acceptor sequence at three distinct sites within the unfolded V_L_ domain (Supplementary Figure S1A). This domain becomes ubiquitinated at multiple sites when the proteasome is inhibited, (Okuda-Shimizu and Hendershot, 2007; Shimizu et al., 2010), making it likely that the VL domain enters the retrotranslocon first. Each of the N-linked glycan consensus sites engineered in the V_L_ domain was readily glycosylated, as shown by comparing the migration of each mutant with the parental NS1 and by Endo H treatment, which cleaved the glycans and restored mobility to that of the non-glycosylated NS1. Inhibition of proteasomal activity with MG132 treatment resulted in a pool that was deglycosylated, even in the case of the NS1-N100 construct, which had the glycan positioned at the V_L_:C_L_ boundary (Figure 1A), revealing that the entire V_L_ domain must reach the cytosol. We next engineered sites in the C_L_ domain and were careful to choose positions within loops of the well-characterized Ig fold, as glycans at these positions should be less likely to interfere with domain folding (Supplementary Figure S1B). All sites were glycosylated and treatment with MG132 also resulted in deglycosylation of a similar amount of each of these modified NS1 constructs (Figure 1B). This indicated that even the well-folded C_L_ domain, which is not ubiquitinated when the proteasome is inhibited, was fully retrotranslocated to the cytosol.

### Deglycosylation is dependent on ERAD substrate ubiquitination and retrotranslocation

**FIGURE 2:**
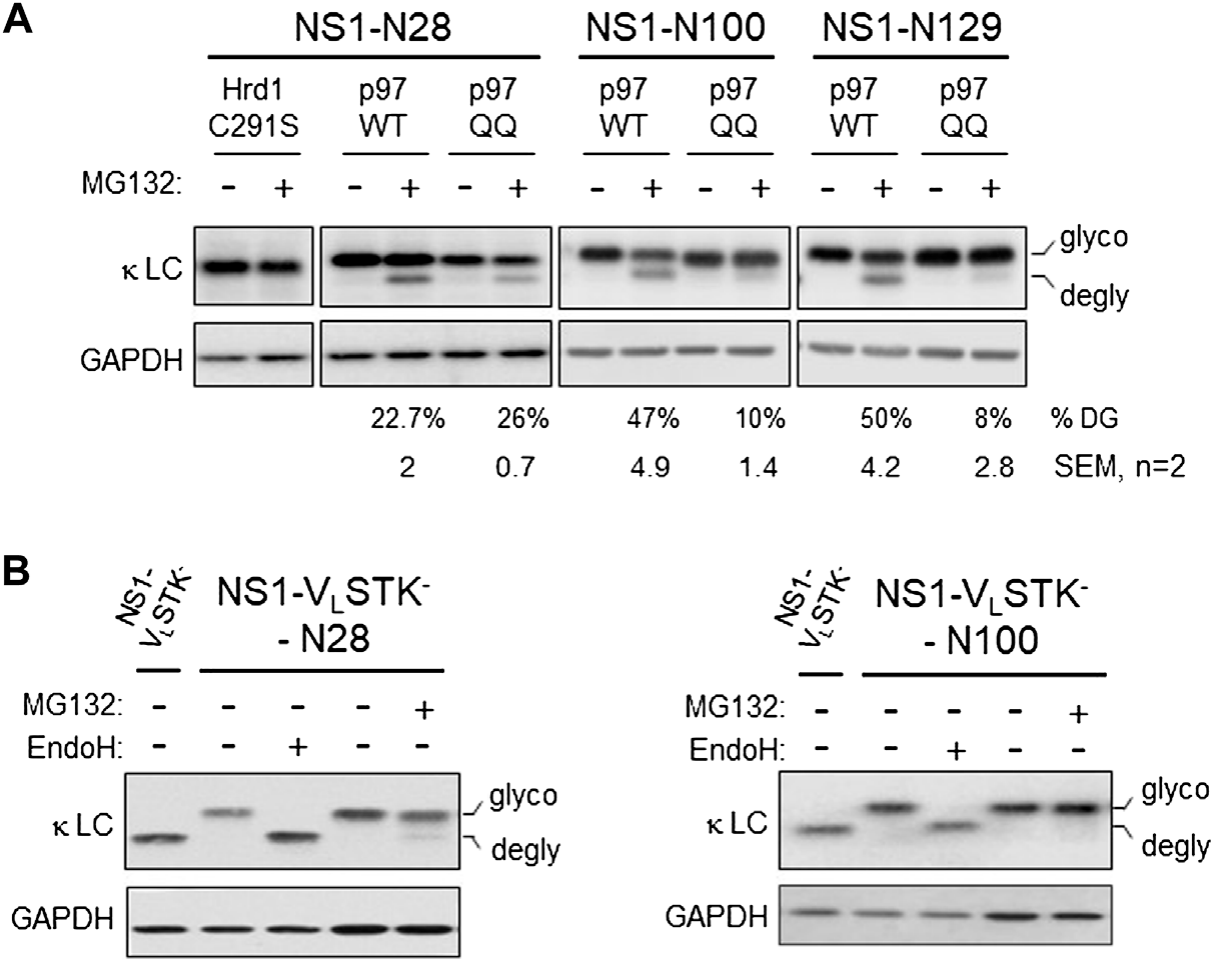
Retrotranslocation of NS1 does not require proteasomal activity but is dependent on ubiquitination and p97. **(A)** Cells were co-transfected with NS1-N28, NS1-N100, or NS1-N129 encoding constructs together with either p97 WT or ATPase inactive p97QQ mutant. The next day, a plate of each was incubated with either 10 μM MG132 (+) or DMSO (- control) for 3.5 h prior to lysis. Lysates were prepared and analyzed by SDS-PAGE coupled western blotting with goat anti-mouse κ antisera or GAPDH as a control. The percent deglycosylation was determined as the fraction of the total amount of LC and is indicated below each lane along with the calculated SEM, *n* = 2. **(B)** Cells were transfected with the NS1-V_L_STK^-^ construct or NS1-V_L_STK^-^ engineered with a single N-linked glycan on the V_L_ domain at N28 or N100. Prior to lysis, one dish of each glycosylated construct was incubated with MG132 (+). Lysates were prepared and one sample of each glycosylated construct was treated with Endo H (+) as indicated. Samples were analyzed as in Figure 1.

Each of the engineered constructs led to nearly complete glycosylation, leading us to conclude the unglycosylated pool present with proteasome inhibition was due to deglycosylation of the pool that was retrotranslocated. Nonetheless, we explored the possibility that the more rapidly migrating band observed was in fact due to MG132 stabilizing a population that never entered the ER but was normally rapidly degraded. Cycloheximide chase experiments were conducted on cells expressing the NS1-N129 construct (Supplementary Figure S2). There was no evidence the more rapidly migrating form appeared with cycloheximide treatment alone. In contrast, when cells were treated with both cycloheximide and MG132 the glycosylated form began to disappear over time, while the deglycosylated form appeared, demonstrating a precursor-product relationship. We next queried whether retrotranslocation, as measured by deglycosylation, was dependent on both ubiquitination and p97 activity. We chose three constructs including one with the engineered glycosylation site present in the V_L_ domain (N28), one at the boundary between the two domains (N100), and one in the C_L_ domain (N129). Co-expression of a domain negative Hrd1 mutant (C291S) inhibited deglycosylaton of the NS1-N28 protein (Figure 2A). These constructs were co-expressed with either wild-type p97 or a dominant negative mutant (QQ) and the effects on deglycosylation of monoglycosylated NS1 proteins was examined when the proteasome was inhibited. Although the mutant p97 inhibited deglycosylation, and thus retrotranslocation, of the N100 and N129 constructs, it had very little effect on the N28 construct. This implies that the V_L_ domain of the NS1 protein can be transported at least this far into the cytosol before the activity of p97 is required to extract this ERAD client.

The lack of client deglycosylation observed with co-expression of the dominant negative Hrd1C291S mutant strongly suggested that ubiquitination was required for retrotranslocation. However, there is evidence that auto-ubiquitination of Hrd1 is critical to its activity in client dislocation *in vitro* (Baldridge and Rapoport, 2016). Hence, we examined a variant of the NS1 LC that cannot be ubiquitinated due to mutation of all lysines, serines, and threonines in the V_L_ domain (NS1-V_L_-STK^-^); the well-folded C_L_ domain is left intact and is not ubiquitinated (Shimizu et al., 2010). A single glycosylation site was engineered near the N-terminus of the unfolded V_L_ domain (N28), and a separate one was introduced near the C-terminus of this domain (N100). In this case, inhibition of the proteasome did not lead to the appearance of a significant amount of the deglycosylated form of either NS1-V_L_-STK^-^ construct (Figure 2B). Thus, client ubiquitination was indeed required for it to be recognized by cytosolic factors, like p97, and pulled far enough into the cytosol for the glycan at N28, or any subsequent glycans, to become accessible to N-glycanase 1.

### Retrotranslocated C_L_ domain is reduced

**FIGURE 3:**
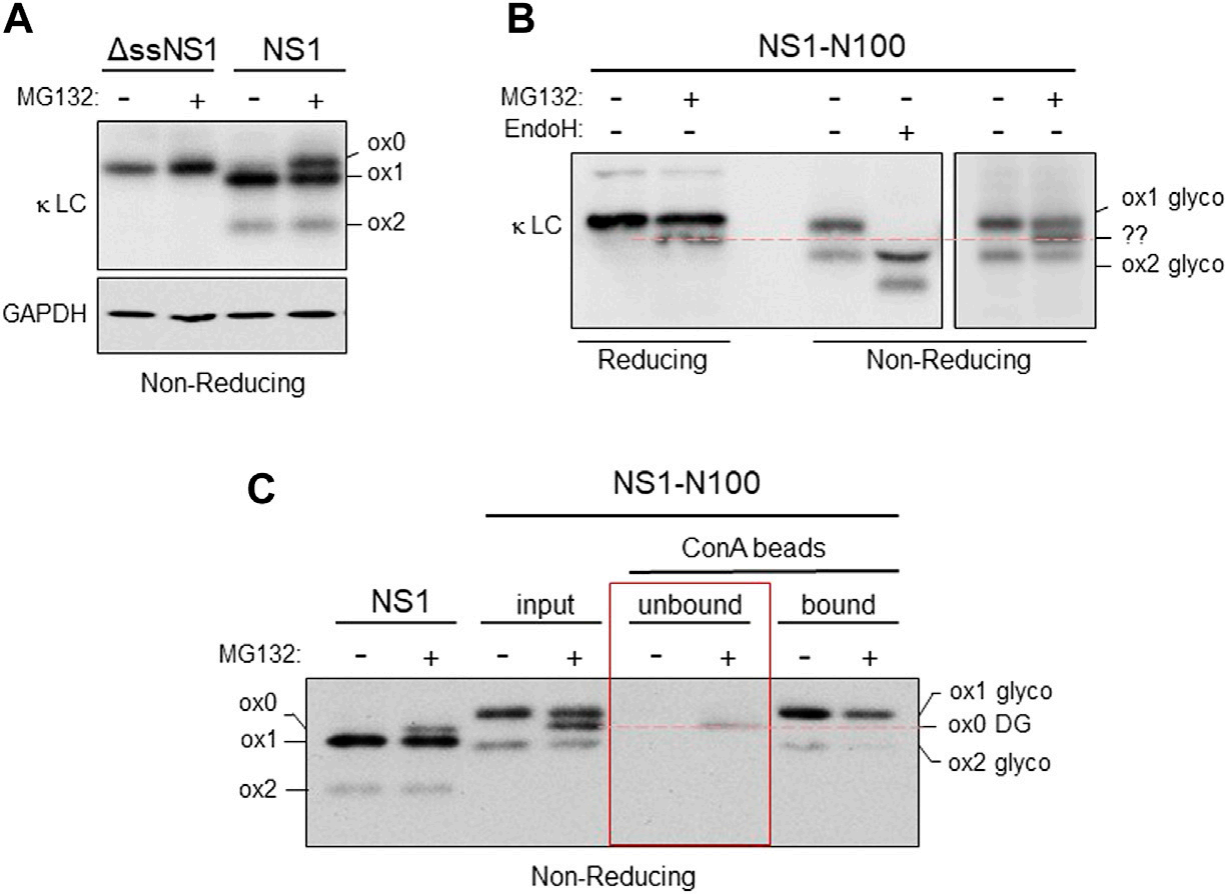
Both domains are reduced in the retrotranslocated NS1 protein. **(A)** Cells were transfected with either the cytosolically expressed ΔssNS1 or NS1. After 24 h, cells were treated with (+) or without (-) MG132, and cell lysates were prepared in NP-40 buffer containing NEM. Samples were analyzed by non-reducing SDS-PAGE-coupled western blotting with goat anti-mouse κ antisera. Migration of the various oxidation species (ox0, ox1, ox2) is indicated. **(B)** Cells expressing NS1-N100 were treated with (+) or without (-) MG132 for 3.5 h prior to lysis. Lysates were prepared and analyzed under reducing or non-reducing conditions as indicated. A portion of the lysate from cells not treated with MG132 was digested with Endo H (+) to determine the migration of the deglycosylated ox1 and ox2 species. An unidentified species (??) generated by proteasomal inhibition observed on non-reducing gels that co-migrated with reduced, deglycosylated NS1-N100 is indicated with a red dotted line. **(C)** 293T cells expressing NS1-N100 were incubated with MG132 (+) or DMSO (-). Cells were lysed in NP-40 lysing buffer supplemented with NEM, and a portion of each was kept as an input. The remaining lysate was absorbed with Con A-conjugated beads. Equivalent fractions of the Con A-unbound and the sample was eluted from the Con A beads (bound) were analyzed by SDS-PAGE conducted under non-reducing conditions and blotted with anti-κ antisera. Bands corresponding to the various redox states are indicated as is their glycosylation status. A red dotted line indicates the species generated by proteasomal inhibition observed panel B is in fact fully reduced and non-glycosylated.

Under steady state conditions, the NS1 protein populates a form in which both domains are oxidized (ox2), and a partially oxidized (ox1) form, in which only the C_L_ domain possesses a disulfide bond (Knittler et al., 1995; Skowronek et al., 1998). The presence of the intramolecular disulfide bond can be detected by increased mobility on non-reducing SDS polyacrylamide gels (Braakman et al., 1992). To determine the redox state of NS1 after proteasome inhibition, we engineered a NS1 κ LC that was devoid of the ER targeting signal sequence (ΔssNS1), which would be synthesized in the cytosol, to serve as a control for the mobility of a completely reduced NS1 protein. When both the ΔssNS1 and the parental NS1 were electrophoresed on the same SDS polyacrylamide gel under non-reducing conditions, we found that in the absence of MG132 NS1 populated two redox forms (ox1 with an oxidized C_L_ domain and ox2 possessing disulfide bonds in both the V_L_ and C_L_ domains), which is consistent with previously reported data (Skowronek et al., 1998). However, when the proteasome was inhibited, we observed an additional slower migrating band that co-migrated with the completely reduced ox0 form observed with the cytosolically expressed ΔssNS1 construct (Figure 3A). This revealed that inhibition of proteasomal degradation generated a pool of NS1 in which both the V_L_ and C_L_ domains were fully reduced. As the fraction of NS1 that was deglycosylated upon proteasomal degradation was similar to the amount that became fully reduced, it was likely that they represented the same pool. To test this directly, we chose the NS1-N100 construct with the single engineered N-glycan in the V_L_ domain. Proteasome inhibition resulted in the appearance of the deglycosylated form when the protein was analyzed under reducing conditions, and the non-MG132 treated sample separates into the ox1 and ox2 forms on non-reducing gels. Endo H treatment increased the mobility of both species, demonstrating that both were glycosylated. When NS1-N100 isolated from non-treated and MG132-treated cells was separated under non-reducing conditions, we observed a third species that migrated between the ox1 and ox2 isoforms found it the proteasome inhibited cells, but slightly slower than the deglycosylated ox1 form generated by Endo H treatment (Figure 3B). To determine if this new species represented reduced and deglycosylated NS1-N100 protein, the glycosylated pool was separated from the deglycosylated one by incubation with Concanavalin A (Con A)-conjugated beads. The Con A lectin binds N-linked glycans, allowing the glycosylated form of the protein to be readily isolated by centrifugation, leaving the deglycosylated form in the supernatant. All samples were electrophoresed under non-reducing conditions and the parental non-glycosylated NS1 was used as a control. The new species observed in proteasome-inhibited cells expressing NS1-N100 co-migrated with the ox0 form of non-glycosylated NS1 and did not bind to Con A (Figure 3C), demonstrating that the new band appearing upon MG132 treatment was deglycosylated and fully reduced.

### Reduction of the C_L_ domain represents a significant rate-limiting step in degradation and retrotranslocation of a κ LC

**FIGURE 4:**
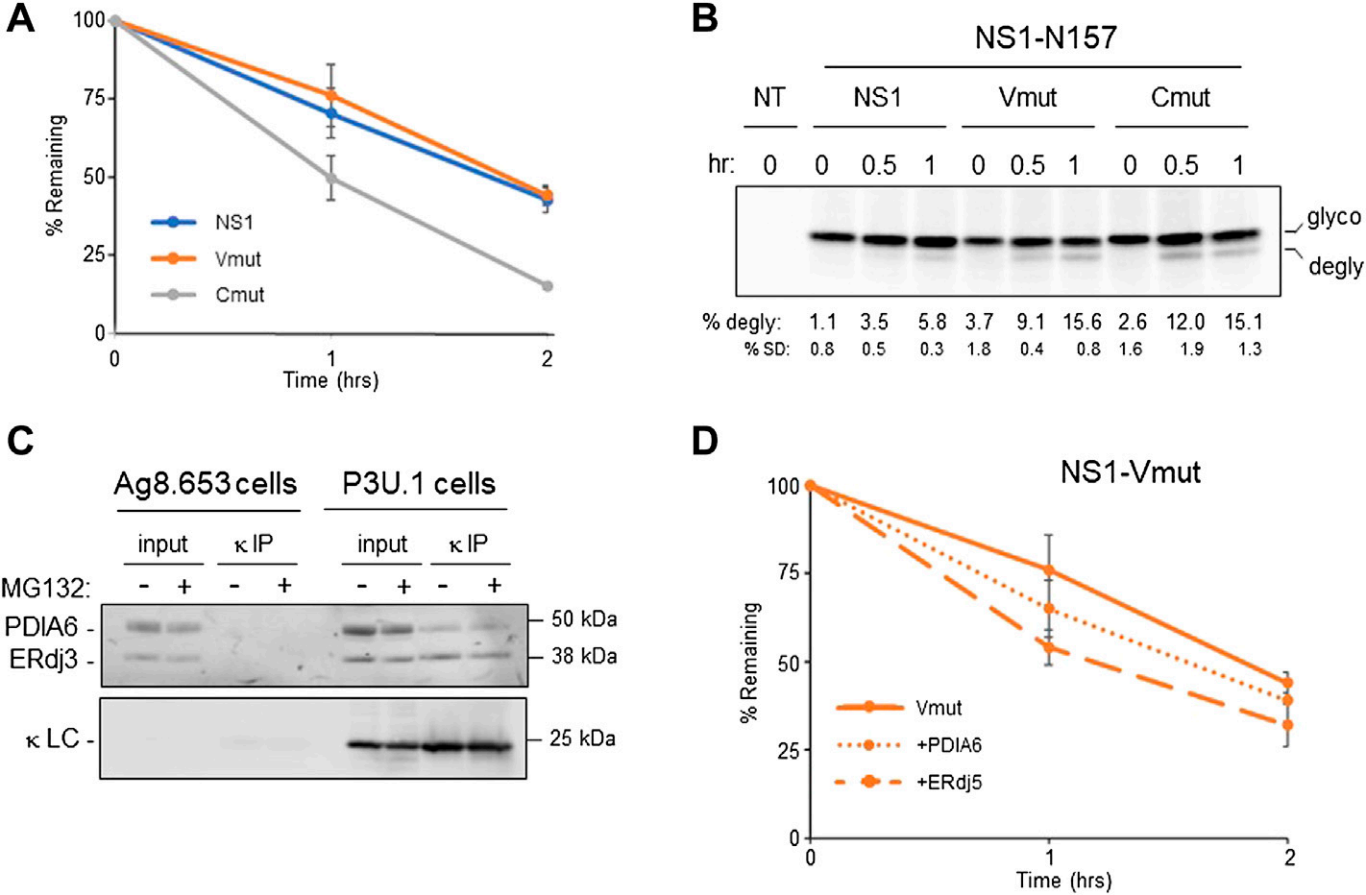
Reduction of the C_L_ domain is a rate-limiting step in retrotranslcoation and degradation. **(A)** Cells expressing parental NS1 or a mutant in which V_L_ (Vmut) or C_L_ (Cmut) cysteines were substituted with serines were pulse-labeled for 15 min and chased as indicated. LC was isolated by immunoprecipitation, electrophoresed, and quantified by Phosphorimaging *n* = 4 for NS1, and *n* = 3 for the V_L_ and C_L_ mutants. Error bars show standard deviation. **(B)** Cells expressing parental NS1-N157 or constructs in which the V_L_ or C_L_ cysteines were substituted were pulse-labeled for 15 min and chased for the indicated times. As MG132 was included in the chase media, cells were incubated with MG132 for between 3 and 4 h prior to lysis depending on the time point harvested. Non-transfected (NT) cells were used to subtract background signals. Percent deglycosylation and standard deviation (SD) are shown below, *n* = 4. **(C)** Ag8.654 (Ig^−^) and P3U.1 (κ^+^) cells were incubated with MG132 (+) or DMSO (-). Prior to lysis, cells were crosslinked with DSP to retain protein:protein interactions. Lysates were prepared, a portion was removed for input, and the remainder was immunoprecipitated with polyclonal anti-κ. Samples were analyzed by reducing SDS-PAGE-coupled western blotting with rabbit anti-PDIA6, rabbit anti-ERdj3, and goat anti-κ. **(D)** The NS1-Vmut was expressed alone or co-expressed with PDIA6 or ERdj5. Cells were pulse-labeled for 15 min and chased as shown. Lysates were prepared, immunoprecipitated with anti-κ and analyzed as in **(A)**, *n* = 3 for V_L_ mutant alone and *n* = 4 for Vmut + PDIA6 and Vmut + ERdj5.

The accumulation of a completely reduced species of NS1 upon inhibition of the proteasome led us to determine how much the reduction of each domain represented an impediment to the retrotranslocation and degradation of this ERAD client. We genetically ablated the disulfide bonds in the V_L_ domain and in the C_L_ domain separately by mutating the two cysteines in each domain to serines (Supplementary Figure S4A) and quantified the effect on the turnover of both constructs using pulse-chase experiments. Although disruption of the disulfide bond in the V domain had very little effect on the half-life of this mutant, disruption of the bond in the C domain significantly increased its turnover (Figure 4A). This revealed that breaking the disulfide bond in the C domain represented a rate-limiting step in the degradation of NS1. In an attempt to determine if this rate-limiting step occurred at the point of retrotranslocation, we employed constructs with a glycan engineered at N157 for the parental NS1 and each of the two domain disulfide mutants. Pulse-chase experiments revealed the glycosylated forms of all three constructs turned over somewhat faster than their non-glycosylated counterparts, perhaps due to their interaction with the lectin chaperones instead of the BiP chaperone complex. Additionally, the glycosylated Vmut possessed a shorter half-life than the glycosylated protein with both disulfide bonds intact, but disruption of the disulfide bond in the constant domain continued to have a greater effect on the turnover of NS1 (Supplementary Figure S4B). MG132-treated cells expressing these monoglycosylated clients were pulse-labeled and shorter chase periods were monitored to catch early time points in retrotranslocation. In keeping with the half-lives of these glycosylated proteins, genetic disruption of the disulfide bond in either domain led to faster retrotranslocation, as judged by deglycosylation, than the NS1 construct possessing both disulfide bonds, whereas differences between the two disulfide mutants was marginal (Figure 4B).

In an attempt to identify the PDI family member(s) responsible for reducing the disulfide bonds in NS1, we used the P3U.1 mouse plasmacytoma cells, which is the source of the NS1 construct used in our studies thus far (Yelton et al., 1978) and the Ag8.653 cells (Kearney et al., 1979), a LC loss variant of P3U.1, as a negative control. P3U.1 cells have been used in multiple studies to examine ERAD requirements for this LC (Knittler et al., 1995; Okuda-Shimizu and Hendershot, 2007). The κ LC were isolated by immunoprecipitation and subjected to mass spectrometric spectral count analysis (Supplementary File S1). Several ER chaperones were identified with the LC, including BiP and GRP94 in keeping with previous reports (Melnick et al., 1992), but since chemical cross-linking was not used to stabilize labile interactions, co-chaperones like ERdj3 or other components of the BiP chaperone complex (Meunier et al., 2002) were not identified. PDIA6 was the only PDI family member found to be associated with NS1 (Table 2 and Supplementary File S1) and has been reported to have reductase activity (Gorasia et al., 2016). To confirm this finding, lysates from P3U.1 and Ag8.653 cells were treated with the cleavable, cross-linking agent DSP, and proteins association with the NS1 κ LC were analyzed by immunoprecipitation-coupled western blotting. Membranes were blotted with antibodies specific for PDIA6, ERdj3 as a positive control, and mouse κ LC (Figure 4C). PDIA6 was detected with the LC in P3U.1 cells, but it was not isolated from κ LC-negative Ag8.653 cells. To determine if PDIA6 was responsible for reducing the C_L_ domain, we expressed the Vmut (possessing only an oxidized C domain) alone or co-expressed it with either PDIA6 or ERdj5, another PDI family member with reductase activity, and performed pulse-chase experiments (Figure 4D). PDIA6 had a modest effect on enhancing the turnover of the NS1 Vmut, but ERdj5 had a greater effect, even though it was not detected in the spectral counts data (Supplementary File S1) or by western blotting (not shown). We also tested the effects of PDIA6 and ERdj5 co-expression on the half-life of the Cmut. PDIA6 had no effect on the turnover rate of Cmut, in keeping with the fact that genetic ablation of the disulfide bond in the V_L_ domain did not change the half-life of the non-glycosylated NS1 LC (Supplementary Figure S4C). Somewhat unexpectedly, co-expression of ERdj5 actually decreased the turnover of the Cmut.

**TABLE 2:** Identification of proteins associated with NS1.

References	SC neg cntrl	SC -MG132	SC + MG132
ER chaperones			
GRP78	2	151	109
GRP94	0	5	2
NS1 LC	0	51	41
CALX	0	5	0
PDIA6	0	2	0
cyto chaperones			
HSP7C	3	26	44
BAG6	0	0	21
UBA52	0	0	53
UPS			
PSA6 (α-core)	0	0	2
PSA7 (α-core)	0	0	2
PRS7 (base)	0	0	2
PRS4 (base)	0	0	2
Cullin-9	0	0	2

### The retrotranslocated, deglycosylated, and fully reduced NS1 C_L_ domain retains structure in the cytosol

**FIGURE 5:**
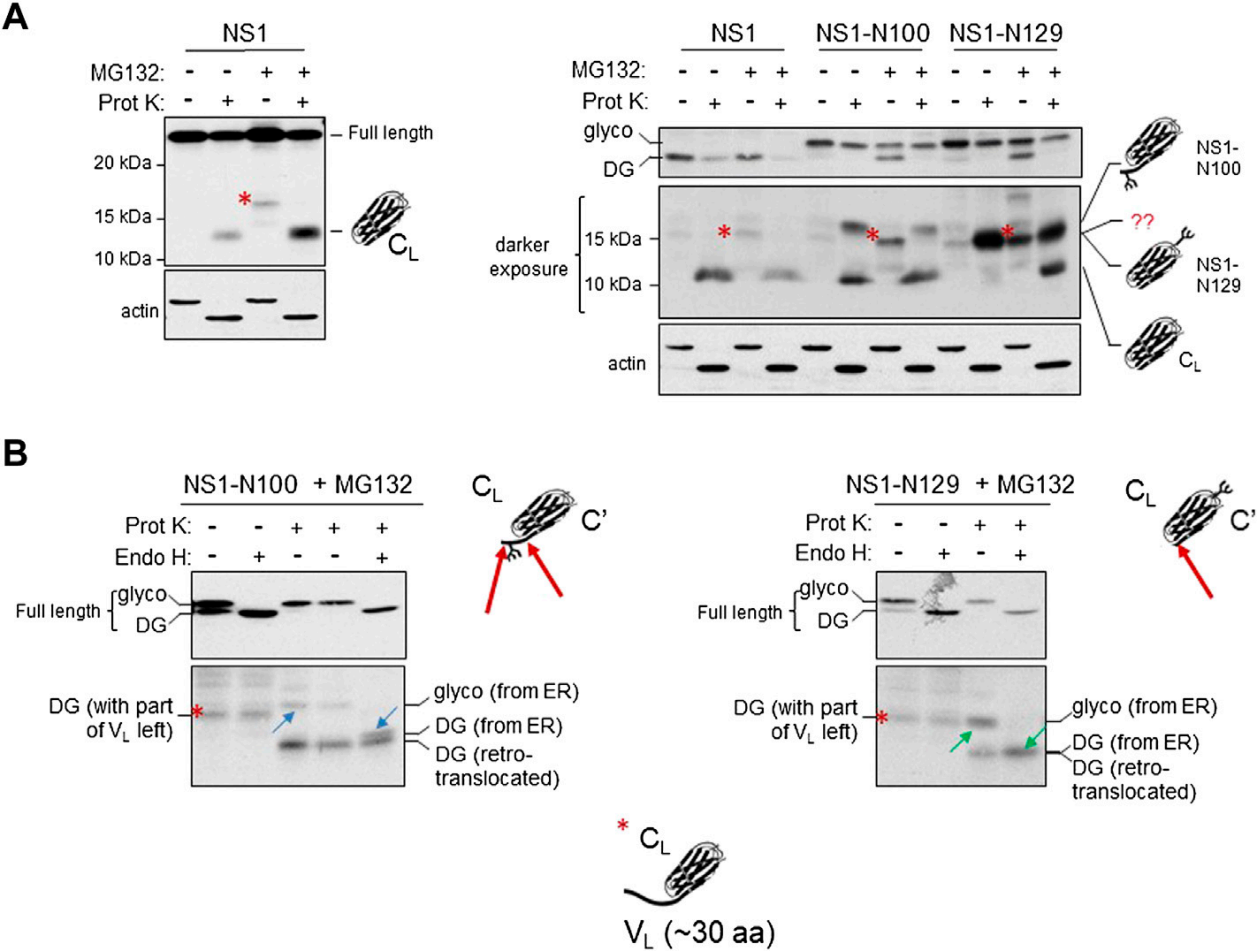
The retrotranslocated and deglycosylated C_L_ domain maintains structure as indicated by resistance to Proteinase K digestion. **(A)** Cells were transfected with parental NS1, NS1-N100 or NS1-N129 and incubated with (+) or without (-) MG132 for 3.5 h prior to lysis. Lysates were treated with (+) or without (-) Proteinase K as described in Materials and Methods and analyzed by western blotting with anti-κ antisera. On the left, analysis of parental NS1 is shown alone, and on the right together with the two glycosylated constructs. Migration of full-length NS1 is shown in the top on right, whereas a longer exposure of the fragments generated is below. A MG132-generated fragment present with all three constructs is indicated with a red asterisk. The deduced identity of the various fragments based on data in **(B)** are portrayed with cartoon schematics. **(B)** Cells expressing the indicated glycosylated constructs were incubated with MG132 prior to lysis. Lysates were treated either with (+) or without (-) Proteinase K, and then split to allow subsequent digestion with Endo H (+) for one half and left undigested (-) for the other half as a control. The altered mobility of fragment pairs that were glycosylated and cleaved by Endo H are indicated with blue arrows for NS1-N100 or green arrows for NS1-N129. Schematic representations of Proteinase K cleavage of both proteins are shown with a red arrow on the schematic. Fragment identities and their source are indicated.

A previous *in vitro* study found that reduction of a recombinant C_L_ domain did not significantly alter its structure (Feige et al., 2007). To determine if the fully reduced species of NS1 expressed in cells still possessed structure, lysates from non-treated or MG132-treated cells expressing the parental NS1 construct were incubated with limiting concentrations of Proteinase K to distinguish between unstructured and structured domains. Samples were electrophoresed on 12% acrylamide gels to resolve fragments of 10–12 kDa. Proteinase K digestion of NS1 from non-MG132-treated lysates (control) yielded a single, readily detectable band migrating with an apparent molecular weight of ∼12 kDa. Importantly, the anti-κ antisera is specific for the constant region, which is consistent with this small fragment representing a complete, well-folded C_L_ domain in which the unstable V_L_ domain had been digested (Figure 5A). When the MG132-treated sample was examined without Proteinase K treatment, we detected a band that migrated somewhat slower than an isolated C_L_ domain (red asterisk). Proteinase K treatment of the lysate from MG132-treated cells resulted in the disappearance of this band and the appearance of an ∼12 kDa band that co-migrated with the band generated in the Proteinase K treated cells in which the proteasome had not been inhibited.

Using this construct, it was not possible to establish if the protected C_L_ band arose only from the ox1 form, which would have been in the ER lumen possessing the intramolecular disulfide bond in the C_L_ domain, or if it also included the retrotranslocated and fully reduced ox0 isoform. To distinguish between these possibilities, we similarly examined lysates from two of the NS1 constructs possessing a single N-linked glycan; the NS1-N100 with an engineered glycan very near the C-terminus of the V_L_ domain and the NS1-N129 with the glycan position in a loop in the C_L_ domain. For the NS1-N100 construct, treatment of the control (no MG132) sample with Proteinase K produced two anti-κ-reactive fragments; both of which must originate from ER localized, glycosylated protein, as this is the only form present without proteasome inhibition (Figure 5A). One of these co-migrated with the Proteinase K protected fragment in the parental non-glycosylated NS1 protein. This likely represented an unglycosylated C_L_ domain generated by proteolytic cleavage at the V_L_:C_L_ boundary just C-terminal of the glycan, however, the source of the slower migrating fragment was not immediately clear (Figure 5A). The same two species were present after Proteinase K digestion of MG132-treated cells, and the non-Proteinase K digested lysate from proteasome-inhibited cells again produced a fragment similar to that found with the parental NS1 protein (red asterisk). For the NS1-N129 construct, a single fragment was produced with protease digestion of lysates from untreated cells (Figure 5A). It migrated faster than the slow migrating band observed in the NS1-N100 protein and co-migrated with the red asterisk band. MG132 treatment alone generated a similar ∼15 kDa fragment as observed with the NS1 and NS1-N100 proteins, and Proteinase K digestion of the MG132-treated samples produced two fragments, including one that co-migrated with the ∼15 kDa band from the non-MG132 treated cells, and the other with the ∼12 kDa band observed with NS1 and NS1-N100.

To determine the source of the various fragments generated in Figure 5A when MG132 treatment was combined with or without Proteinase K digestion, lysates were further digested with endoglycosidase H (Endo H) to remove the glycan if present. In the case of the NS1-N100 construct, Endo H treatment of the MG132 lysate that was not digested with Proteinase K demonstrated the fragment (red asterisk) was not glycosylated and therefore arose from a cytosolic pool of this protein (Figure 5B). Lysates from the proteasome-inhibited cells that had been treated with Proteinase K were further digested with Endo H. This revealed the slower migrating band (blue upward arrow) was glycosylated (derived from ER) but collapsed to a band slightly larger than the isolated C_L_ domain (blue downward arrow), whereas the fastest migrating fragment in this sample was unglycosylated and thus represented the retrotranslocated protein that had been deglycosylated by N-glycanase and was digested by Proteinase K to an isolated C_L_ domain. Thus, it appeared that the glycan engineered just 9 amino acids from the V_L_:C_L_ boundary partially interfered with complete proteolysis of the V_L_ domain (indicated with two red arrows on the cartoon) giving rise to the doublet in the Proteinase K and Endo H treated sample.

When the NS1-N129 construct was similarly examined (Figure 5B), we again found that Endo H digestion of the sample from MG132 treated cells that was not treated with Proteinase K revealed that the MG132-generated fragment (red asterisk) was not glycosylated and therefore arose from a cytosolic pool of this protein. The Proteinase K-treated sample from this glycosylated construct had produced two fragments, and Endo H digestion revealed that the slowest migrating band (green upward arrow) was glycosylated and collapsed to the non-glycosylated faster migrating C_L_ domain (green downward arrow) after the glycan was removed. In this case the position of the engineered glycan did not interfere with complete proteolysis of the V_L_ domain. Furthermore, the glycosylated, isolated C_L_ domain (green upward arrow) was shown to co-migrate with the MG132-generated fragment (red asterisk). Cartoon depictions of the composition of the various bands generated as deduced from the combinations of treatments are included in the figure and Supplementary Figure S5. Together, the data presented in this figure strongly indicated that the C_L_ domain of the deglycosylated ox0 isoform generated after MG132 treatment still possessed significant structure in the cytosol, even though the disulfide bond had been reduced. Based on the molecular weight of the MG132-generated, non-glycosylated fragment recognized by anti-κ LC antisera we deduced that it must include ∼30 amino acids of the V_L_ domain in addition to the 105 amino acid C_L_ domain and represents an intermediate in proteasomal degradation.

### Retrotranslocated NS1 LC were associated with regulatory particle ATPases and core subunits of the proteasome when degradation was inhibited

**FIGURE 6:**
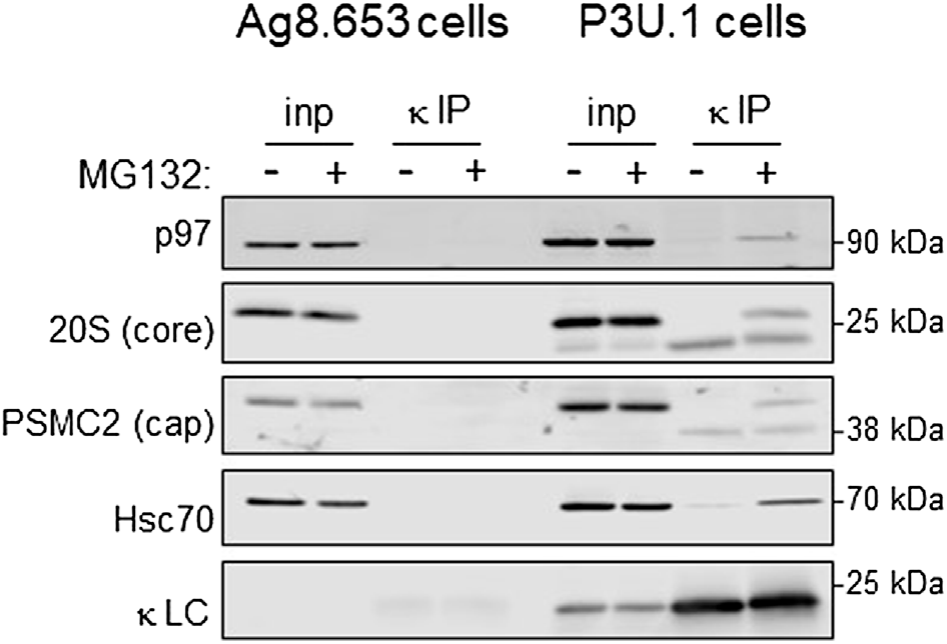
Proteasomal inhibition results in the association of retrotranslocated κ LCs with AAA-ATPase base and 20S core subunits. **(A)** P3U.1 (κ^+^) and Ag8.653 (Ig^−^) cells were incubated with MG132 (+) or DMSO (-) for 3.5 h. Prior to lysis, cells were crosslinked with DSP to retain protein:protein interactions. Lysates were prepared using RIPA buffer; a portion was removed for input, and the remainder was immunoprecipitated with anti-LC-conjugated agarose beads. Immunoprecipitated material (IP) and total lysate samples (input) were separated on reducing SDS-gels and analyzed by western blotting with an anti-p97, anti-20S proteasome core α subunit, anti-PSMC ATPase regulatory particle subunit, anti-Hsc70, or anti-κ LC.

**FIGURE 7:**
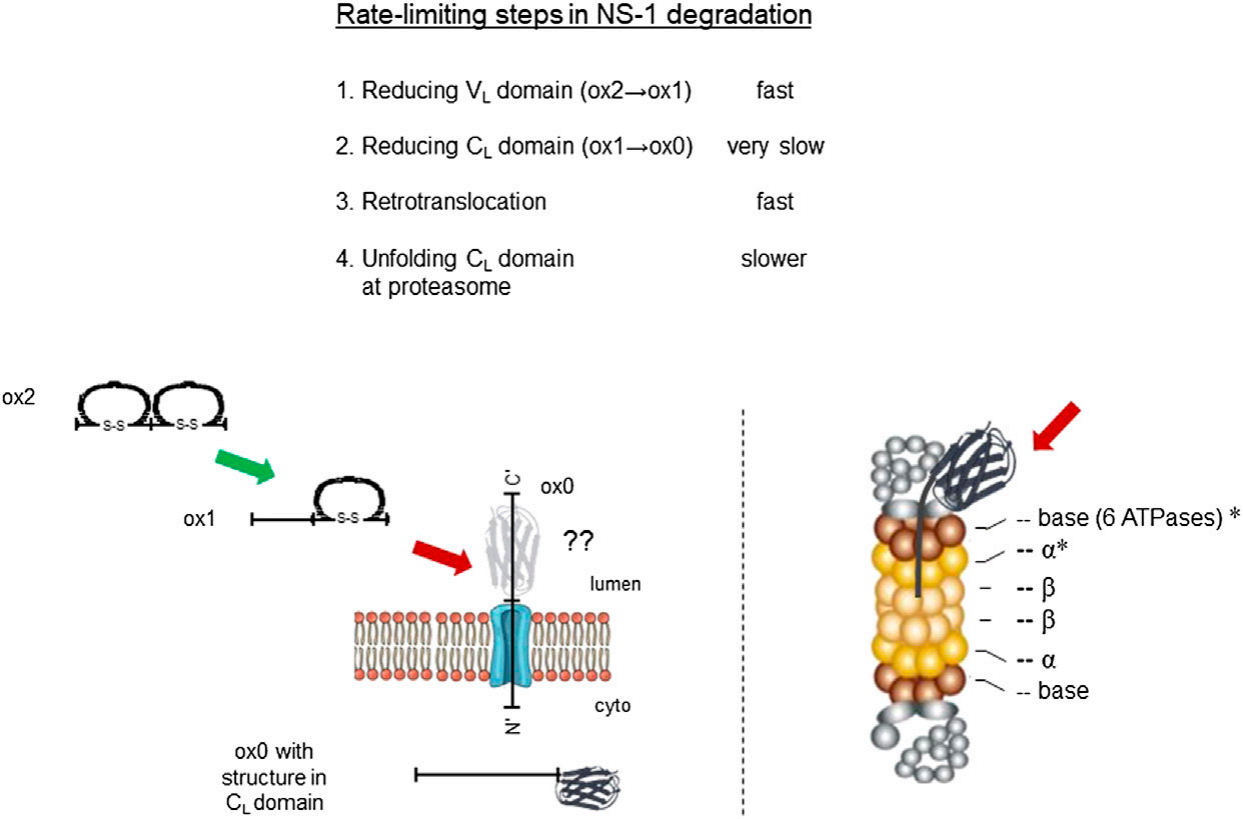
Identification of rate-limiting steps in NS1 degradation. Delineation of degradation steps revealed in this study and their relative rate of execution are shown at top. Schematic of steps in the reduction and retrotranslocation of NS1 on left, and the location of proteasomal subunits engaging the MG132-generated fragment are shown with asterisks on the right. Fast steps are indicated with a green arrow and slower ones with red arrows. The unknown conformation of the reduced C_L_ domain as it passes through the retrotranslocon is portrayed in grey with a question mark.

To identify proteins that the retrotranslocated NS1 protein interacted with, we examined the spectral count data that was obtained with κ LC immunoprecipitated from the P3U.1 cells obtained with and without inhibition of the proteasome (Table 2 and Supplementary File S1). We identified several proteasome subunits associated with the LC only after MG132 treatment, including two core components of the 20S proteosome and two ATPase subunits of the proteasome regulatory particle. To validate this finding, anti-κ immunoprecipitated proteins were isolated from Ag8.653 and P3U.1 cells that were pre-treated with or without MG132 and processed for western blotting. We found that κ LCs associated with p97, regulatory subunit 7 (PSMC2) of the ATPase ring component of the proteasome cap, and the α7 subunit of the proteasome core when MG132 was used, but not in its absence, nor were they detected in anti-κ isolated material from the control Ag8.653 cells (Figure 6). In combination, these data indicate that a portion of the retrotranslocated NS1 LCs possessing a constant domain that retains structure are stably bound to the proteasome when its proteolytic activity was inhibited. Given the known dimensions of the proteasome (Rape and Jentsch, 2002), the distance from the active proteolytic sites to the outside of the 20S core would require ∼20 amino acids of an extended polypeptide chain and another 10–15 to cross the ATPase ring. The ∼15 kDa anti-κ-reactive fragment that we observed in proteasome inhibited cells was calculated to possess ∼30–35 additional amino acids of the V_L_ domain, which would be sufficient to reach the proteolytic active site, indicating that unfolding of the structured C_L_ domain by the ATPase ring of the proteasome represented a second rate-limiting step in the degradation of NS1 κ LCs (Figure 7).

## Discussion

Ig HC are expressed first in pre-B cells during B cell development, and the association of BiP with the unstructured C_H_1 domain ensures the free HC are not secreted. Subsequently, LC rearrangement is initiated to produce a single LC that must induce folding of the C_H_1 domain and release BiP, so the assembled Ig protein can be transported to the cell surface or secreted. This represents a critical checkpoint in both B cell development and in Ig assembly, and as such, LC genes have evolved to produce C_L_ domains that fold very stably. The failure of a LC to rescue the free HC can initiate another round of LC gene rearrangements, necessitating the disposal of the LC produced first. Even once a compatible LC partner has been selected, the extremely high rate of antibody synthesis observed in plasma cells is likely to result in some failures in proper Ig maturation resulting in the need to target improperly folded subunits for degradation. The NS1 LC is an example of such a protein and provides an excellent model to ask what the requirements and checkpoints for degrading a protein when one domain fails to fold, and the other domain folds very stably and secured with a disulfide bond. Our studies revealed two checkpoints or rate-limiting steps in the degradation of this non-secreted LC (Figure 7). The first is the reduction of the C_L_ domain. Although the V_L_ domain of NS1 obtains a disulfide bond initially, it is readily reduced, due to its unstable fold giving rise to the ER resident ox1 form of NS1. Although we observed PDIA6 associated with NS1, over-expression of this reductase only modestly increased the turnover of the Vmut construct in which the only disulfide bond present was in the C_L_ domain. PDIA6 over-expression had even less of an effect on the half-life of the Cmut that possessed only the V_L_ domain disulfide bond. The interpretation of this result is complicated by the fact that genetically ablating the disulfide bond in the V_L_ domain is not rate-limiting for NS1 turnover. Thus, it remains formally possible that PDIA6 plays a role in reducing the disulfide bond in the V_L_ domain. Although we were unable to detect endogenous ERdj5 association with NS1, perhaps because its association is too transient to detect in these types of experiments, over-expression of ERdj5 readily increased the turnover of the Vmut construct arguing that it is responsible for reducing the disulfide in the C_L_ domain and thus executing this rate-limiting step. Upon reduction of the C_L_ domain, this LC is retrotranslocated and degraded so rapidly that a completely reduced, ox0 form is never observed unless proteasomal activity is inhibited. These data argue that accessibility of this PDI family member to the disulfide bond in the C_L_ domain is restricted by the very stable fold of this domain.

The extraction of the NS1 LC from the ER lumen required the activity of the AAA-ATPase p97 but was not dependent on proteasomal function. A group of papers from the Sitia lab has examined requirements for degradation of Ig μ heavy chain multimers (Mancini et al., 2000) and μ heavy chain-TCRα chimeras (Fagioli et al., 2001), both of which possess multiple folded domains and are covalently assembled with subunits making them very complicated ERAD clients. In both cases, interchain disulfides were shown to be reduced prior to retrotranslocation. It is noteworthy that proteasome inhibition resulted in reduction of the inter- and intra-molecular disulfide bonds of the J chain that is covalently bound to the μ chain multimers and deglycosylation of this subunit, indicating that it was dislocated to the cytosol. However, the freed μ heavy chain multimers showed no evidence of deglycosylation, arguing that this dimeric ERAD client with oxidized domains at both its N- and C-termini was not retrotranslocated in the absence of proteasomal function (Mancini et al., 2000).

Our data on the requirements for ERAD components in the extraction of NS1 from the ER are contrary to a study using proximity biotinylation, which found that this client was modified by cytosolically expressed BirA even in the presence of the p97QQ mutant, as was the NS1 V_L_-STK^-^ mutant that cannot be ubiquitinated (Sasset et al., 2015). Although this group found that the co-expression of a BiP trap mutant, which is not released from the client, and therefore upstream of retrotranslocation, did prevent biotinylation. Based on our data using these same mutants and constructs, it is possible that the proximity labeling experiments might be revealing continuous sampling of the cytosol by the termini of ERAD clients, which would require that BiP be released. In support of this possibility, an earlier study revealed that BiP release coincided with client retrotranslocation (Chillaron and Haas, 2000).

Somewhat less expected was our finding that, in spite of being reduced for retrotranslocation, the C_L_ domain either retains or re-obtains structure in the cytosol. Indeed, previous studies from our lab revealed that light chain constant domains fold so rapidly in the ER that BiP trap mutants are unable to bind and prevent their folding and are more resistant to heat/chemical denaturation *in vitro* than the molecular chaperone BiP (Hellman et al., 1999). However, the observation raises the question as to the folded state of this LC as it passes through the retrotranslocon. Although the dimensions of actively translocating retrotranslocons in cells have not been determined, there have been several studies that provide insights into this point. The addition of EGFP to an ERAD client revealed that fluorescence was retained throughout retrotranslocation, arguing that either it was not unfolded during extraction or that it refolded very rapidly (Fiebiger et al., 2002). In another study, DHFR was tethered to the N-terminus of the MHC Class I protein, which is an ERAD client in the absence of assembly with β_2_ microglobulin. They found that addition of methotrexate to stabilize the DHFR moiety in a fully folded state did not impede retrotranslocation (Tirosh et al., 2003). The narrowest cross-section of DHFR is 40 Å, and the dimensions of an Ig domain are 40Å × 25Å × 25Å, suggesting this domain might be able to pass through the channel with some structure intact. A cryo-EM structure obtained for yeast Hrd1 dimers complexed with Hrd3, revealed a very narrow channel (Schoebel et al., 2017), which would be incompatible with proteins retaining structure as they passed through the retrotranslocon. However, additional proteins like Der1, Usa1, and Yos9 are known to be components of retrotranslocons in yeast and mammal, which could alter the dimensions of the channel. Ig domains are comprised of 7-9 antiparallel β strands, in which strands 1-4 form one face of the structure and are disulfide bonded to the second part of the structure comprised of strands 5-7 with a Greek key topology (Bork et al., 1994). It is conceivable that reduction of the bond between strands 2 and 6 inside the ER could allow sufficient unfolding to separate the two portions of the domain, allowing it to pass through a narrower channel while retaining enough structure to refold in the cytosol. A better understanding of retrotranslocon dimensions during the extraction process is needed to determine the limitations on client structure.

The continued presence of a structured domain while this ERAD client was in the cytosol provided the second rate-limiting step in its turnover. Intriguingly, our studies detected a very sharp, anti-κ reactive intermediate migrating at ∼15–16 kDa when MG132 was used without the addition of a protease. This fragment is a minor population and small enough that it runs near the dye front and is readily lost if the samples are electrophoresed to long. Based on the apparent molecular weight of this species together with the actual sequence of the NS1 protein, we calculated that this fragment was comprised of the ∼12 kDa C_L_ domain together with an additional ∼30 amino acids of the V_L_ domain. It has been estimated that the distance from the outside of the 20S proteasome to the proteolytic active site is ∼70Å (Hoppe et al., 2000), which is equivalent to ∼20 amino acids of an extended polypeptide chain and is consistent with the C_L_ domain stalling ∼10 amino acids from the 20S proteasome particle. This argues that the C_L_ domain represented an impediment to entry into the proteasome core, consistent with this domain retaining structure, until it was unfolded by the AAA-ATPases found in the base of the proteasome lid (de la Pena et al., 2018). This interpretation is in keeping with our finding that two of the ring ATPases and two of the α subunits of the core 20S proteasome co-immunoprecipitated with NS1.

In summary, our studies revealed two checkpoints in the degradation of an Ig LC. First, the disulfide bond in the well-folded C_L_ domain must be broken for the LC to be transported to the cytosol. In spite of becoming reduced, this domain retains or re-obtains structure in the cytosol. This structured domain represents the second rate-limiting step, and it must be unfolded by the AAA-ATPases that are a component of the proteasome regulatory particle. Since all κ LC share the C_L_ domain, these checkpoints are valid for any LC that fails to achieve its native state. Although our studies focused on a non-secreted κ LC, the Ig fold is the second most common structural motif found in metazoan proteins and is particularly abundant in cell surface receptors and secreted proteins (Muller et al., 2002). This due to a combination of the stability of Ig fold, which is secured by an intradomain disulfide bond, and the ready ability to modify the 8 loops connecting the β strands that comprise the domain. Thus, insights gained on the checkpoints for disposal of this LC are likely to be relevant for the degradation of other proteins possessing an Ig fold(s) that fail to reach their native state.

## Data Availability

The raw data supporting the conclusions of this article will be made available by the authors, without undue reservation.
