# Supplementary material for: Identification of two rate-limiting steps in the degradation of partially folded immunoglobulin light chains
Source: Front Cell Dev Biol. 2022 Aug 22;10:924848. doi: 10.3389/fcell.2022.924848 (PMC9441772; doi:10.3389/fcell.2022.924848)
Supplement: Supplementary file 1 [file Table1.pdf]

St. Jude Children's Research Hospital
Proteomics Facility

http://home.stjude.org/proteomics-mass-spectrometry/Pages/default.aspx

Project name: Identification of proteins interacting with NS1
Service: Proteome profiling by spectral counting (SC)
PI: Dr. Linda Hendershot
User: Melissa Mann
Date submitted: 5/9/2019
Date reported: 5/14/2019
Facility staff (primary): Pagala, Vishwajeeth
Facility staff (secondary): Kavdia, Kanisha

Results (see other spreadsheets by clicking the tabs at the bottom left corner on this page)

Table 1. Summary of the MS analysis

Samples	Total Identified Proteins	Total Spectral Counts
NegControl	13	42
-MG132	28	332
+MG132	36	347

Note: the sequences of identified peptides are available upon request.

NegCntrl vs -MG132- Spectral count comparison of proteins identified from the NegCntrl and -MG132 samples
NegCntrl vs +MG132- Spectral count comparison of proteins identified from the NegCntrl and +MG132 samples
NegMG132 vs +MG132- Spectral count comparison of proteins identified from the NegMG132 and +MG132 samples
Figure 1. Stained SDS Gel image of submitted samples

Discussion

1. Spectral count, a semi-quantitative index, is the total number of MS/MS spectra matched to an assigned protein.
2. For the immunoprecipitation (IP) analysis, the bait protein needs to be among the top ten abundant proteins to improve the possibility of detecting its interacting proteins.

Attention: this table may include multiple pages.

NegCntrl vs -MG132- Spectral count comparison of proteins identified from the NegCntrl and -MG132 samples

SC: the summed number of spectral counts (i.e. MS/MS scans) assigned to one identified protein; TP: total number of peptides identified for a protein; Group: Identified proteins that share one or more peptides are grouped together (e.g. 01). The group is represented by the protein with maximal spectra count (e.g. 01.1). If other proteins in the same group are assigned with at least one uniquely identified peptides, these proteins are also counted as unique proteins (e.g. 01.2); Abundance: SC x 50 (KD) / protein size (KD). p value less than 0.05 generally indicates significant change. The p value is derived by G-test (Zhou JY, Afjehi-Sadat L, Asress S, et al. J Proteome Res. 2010, 9:5133-41). The summed SC of the control samples was compared with that of the Test samples.

Group	Reference	Description	SC NegCntrl	SC -MG123	TP NegCntrl	TP -MG123	Mass (KD)	Abundance	p-value
109198.1	sp|P20029|GRP78_MOUSE	78 kDa glucose-regulated protein OS=Mus musculus GN=Hspa5 PE=1 SV=3	2	151	2	39	72	180.99	2.14806E-43
100001.1	cu|NS1_Cus|NS1_Cus|Light	chain Custom sequence	0.1	51	0	11	27	171.16	8.06923E-17
109199.1	sp|P63017|HSP7C_MOUSE	Heat shock cognate 71 kDa protein OS=Mus musculus GN=Hspa8 PE=1 SV=	3	26	2	11	71	51.53	4.80855E-06
109195.1	sp|P17156|HSP72_MOUSE	Heat shock-related 70 kDa protein 2 OS=Mus musculus GN=Hspa2 PE=1 SV	0.1	15	0	5	70	22.99	8.91784E-06
105429.1	sp|P17182|ENOA_MOUSE	Alpha-enolase OS=Mus musculus GN=Eno1 PE=1 SV=3	0.1	13	0	6	47	21.23	3.76529E-05
114887.1	sp|P52480|KPYM_MOUSE	Pyruvate kinase PKM OS=Mus musculus GN=Pkm PE=1 SV=4	0.1	12	0	7	58	17.3	7.76021E-05
109192.1	sp|Q61696|HS71A_MOUSE	Heat shock 70 kDa protein 1A OS=Mus musculus GN=Hspa1a PE=1 SV=2	0.1	11	0	4	70	13.56	0.000160328
109194.1	sp|P16627|HS71L_MOUSE	Heat shock 70 kDa protein 1-like OS=Mus musculus GN=Hspa1l PE=2 SV=4	0.1	10	0	4	71	12.75	0.000332193
119792.1	sp|P17751|TPIS_MOUSE	Triosephosphate isomerase OS=Mus musculus GN=Tpi1 PE=1 SV=4	0.1	9	0	5	32	13.99	0.000690662
105211.1	sp|P10126|EF1A1_MOUSE	Elongation factor 1-alpha 1 OS=Mus musculus GN=Eef1a1 PE=1 SV=3	0.1	8	0	5	50	14.98	0.001441968
110677.1	sp|P06151|LDHA_MOUSE	L-lactate dehydrogenase A chain OS=Mus musculus GN=Ldha PE=1 SV=3	0.1	7	0	4	36	13.71	0.003026258
117247.1	sp|O70456|1433S_MOUSE	14-3-3 protein sigma OS=Mus musculus GN=Sfn PE=1 SV=2	7	0.1	2	0	28	12.64	0.003026258
105507.1	sp|Q8CGC7|SYEP_MOUSE	Bifunctional glutamate/proline--tRNA ligase OS=Mus musculus GN=Eprs PE	0.1	6	0	2	170	3.82	0.006393648
109186.1	sp|P11499|HS90B_MOUSE	Heat shock protein HSP 90-beta OS=Mus musculus GN=Hsp90ab1 PE=1 SV:	0.1	6	0	5	83	6.01	0.006393648
102600.1	sp|P35564|CALX_MOUSE	Calnexin OS=Mus musculus GN=Canx PE=1 SV=1	0.1	5	0	4	67	3.72	0.01362796
109187.1	sp|P08113|ENPL_MOUSE	Endoplasmin OS=Mus musculus GN=Hsp90b1 PE=1 SV=2	0.1	5	0	4	92	3.79	0.01362796
120283.1	sp|P99024|TBB5_MOUSE	Tubulin beta-5 chain OS=Mus musculus GN=Tubb5 PE=1 SV=1	0.1	5	0	2	50	11.08	0.01362796
120124.1	tr|Q9R0T7|Q9R0T7_MOUSE	MCG15085 OS=Mus musculus GN=Try4 PE=2 SV=1	5	0.1	1	0	26	9.52	0.01362796
108974.1	sp|P62806|H4_MOUSE	Histone H4 OS=Mus musculus GN=Hist1h4a PE=1 SV=2	4	0.1	2	0	11	17.6	0.0294083
109978.1	sp|Q02257|PLAK_MOUSE	Junction plakoglobin OS=Mus musculus GN=Jup PE=1 SV=3	4	0.1	3	0	82	2.45	0.0294083
117019.1	sp|Q8K2B3|SDHA_MOUSE	Succinate dehydrogenase [ubiquinone] flavoprotein subunit, mitochondria	4	12	3	6	73	13.1	0.040759517
109258.2	sp|P03975|IGEB_MOUSE	IgE-binding protein OS=Mus musculus GN=Iap PE=2 SV=1	0.1	3	0	1	63	5.58	0.064646504
109719.1	sp|P24547|IMDH2_MOUSE	Inosine-5'-monophosphate dehydrogenase 2 OS=Mus musculus GN=Impdh	0.1	3	0	1	56	6.27	0.064646504
116659.1	sp|P62908|RS3_MOUSE	40S ribosomal protein S3 OS=Mus musculus GN=Rps3 PE=1 SV=1	0.1	3	0	2	27	11.25	0.064646504
120282.1	sp|P68372|TBB4B_MOUSE	Tubulin beta-4B chain OS=Mus musculus GN=Tubb4b PE=1 SV=1	0.1	3	0	1	50	5.02	0.064646504
105969.1	sp|Q14BJ1|FA89A_MOUSE	Protein FAM89A OS=Mus musculus GN=Fam89a PE=2 SV=2	3	0.1	1	0	19	8.03	0.064646504
100058.1	tr|Q14AA6|Q14AA6_MOUSE	MCG49183 OS=Mus musculus GN=1700009N14Rik PE=2 SV=1	0.1	2	0	2	24	4.11	0.146611857
101875.1	sp|Q03265|ATPA_MOUSE	ATP synthase subunit alpha, mitochondrial OS=Mus musculus GN=Atp5a1 I	0.1	2	0	2	60	4.19	0.146611857
105555.1	sp|Q91X78|ERLN1_MOUSE	Erlin-1 OS=Mus musculus GN=Erlin1 PE=1 SV=1	0.1	2	0	1	39	2.57	0.146611857
106570.1	sp|P16858|G3P_MOUSE	Glyceraldehyde-3-phosphate dehydrogenase OS=Mus musculus GN=Gapdh	0.1	2	0	2	36	6.99	0.146611857
109185.1	sp|P07901|HS90A_MOUSE	Heat shock protein HSP 90-alpha OS=Mus musculus GN=Hsp90aa1 PE=1 SV	0.1	2	0	2	85	2.36	0.146611857
114565.1	sp|Q922R8|PDIA6_MOUSE	Protein disulfide-isomerase A6 OS=Mus musculus GN=Pdia6 PE=1 SV=3	0.1	2	0	2	48	2.08	0.146611857
118320.1	sp|Q64337|SQSTM_MOUSE	Sequestosome-1 OS=Mus musculus GN=Sqstm1 PE=1 SV=1	0.1	2	0	2	48	5.19	0.146611857
105319.1	sp|P60843|IF4A1_MOUSE	Eukaryotic initiation factor 4A-I OS=Mus musculus GN=Eif4a1 PE=1 SV=1	2	0.1	1	0	46	2.17	0.146611857
108023.1	tr|B2RXM2|B2RXM2_MOUSE	EG627828 protein OS=Mus musculus GN=Gm6793 PE=2 SV=1	2	0.1	1	0	37	5.47	0.146611857
110477.1	sp|Q99M73|KRT84_MOUSE	Keratin, type II cuticular Hb4 OS=Mus musculus GN=Krt84 PE=2 SV=2	2	0.1	1	0	65	1.54	0.146611857
114894.1	sp|P97350|PKP1_MOUSE	Plakophilin-1 OS=Mus musculus GN=Pkp1 PE=2 SV=1	2	0.1	1	0	81	1.24	0.146611857
120278.1	sp|Q7TMM9|TBB2A_MOUSE	Tubulin beta-2A chain OS=Mus musculus GN=Tubb2a PE=1 SV=1	2	3	2	1	50	7.02	0.653629236

Attention: this table may include multiple pages.

NegCntrl vs +MG132- Spectral count comparison of proteins identified from the NegCntrl and +MG132 samples

SC: the summed number of spectral counts (i.e. MS/MS scans) assigned to one identified protein; TP: total number of peptides identified for a protein; Group: Identified proteins that share one or more peptides are grouped together (e.g. 01). The group is represented by the protein with maximal spectra count (e.g. 01.1). If other proteins in the same group are assigned with at least one uniquely identified peptides, these proteins are also counted as unique proteins (e.g. 01.2); Abundance: SC x 50 (KD) / protein size (KD). p value less than 0.05 generally indicates significant change. The p value is derived by G-test (Zhou JY, Afjehi-Sadat L, Asress S, et al. J Proteome Res. 2010, 9:5133-41). The summed SC of the control samples was compared with that of the Test samples.

Group	Reference	Description	SC NegCntrl	SC +MG123	TP NegCntrl	TP +MG123	Mass (KD)	Abundance	p-value
109198.1	sp|P20029|GRP78_MOUSE	78 kDa glucose-regulated protein OS=Mus musculus GN=Hspa5 PE=1 SV=3	2	109	2	36	72	180.99	5.89411E-31
108080.1	tr|J3QK04|J3QK04_MOUSE	MCG67952 OS=Mus musculus GN=Gm7808 PE=4 SV=1	0.1	53	0	2	15	180.79	1.98692E-17
107863.1	tr|D3YYZ2|D3YYZ2_MOUSE	MCG1031578 OS=Mus musculus GN=Gm5239 PE=4 SV=1	0.1	51	0	2	14	181.31	8.06923E-17
100001.1	cu|NS1_Cus|NS1_Cus|Light	chain Custom sequence	0.1	41	0	9	27	171.16	9.00418E-14
109199.1	sp|P63017|HSP7C_MOUSE	Heat shock cognate 71 kDa protein OS=Mus musculus GN=Hspa8 PE=1 SV=	3	44	2	16	71	51.53	5.93345E-11
102095.1	sp|Q9Z1R2|BAG6_MOUSE	Large proline-rich protein BAG6 OS=Mus musculus GN=Bag6 PE=1 SV=1	0.1	21	0	12	121	8.68	1.22518E-07
109195.1	sp|P17156|HSP72_MOUSE	Heat shock-related 70 kDa protein 2 OS=Mus musculus GN=Hspa2 PE=1 SV	0.1	17	0	6	70	22.99	2.12577E-06
109192.1	sp|Q61696|HS71A_MOUSE	Heat shock 70 kDa protein 1A OS=Mus musculus GN=Hspa1a PE=1 SV=2	0.1	8	0	4	70	13.56	0.001441968
109194.1	sp|P16627|HS71L_MOUSE	Heat shock 70 kDa protein 1-like OS=Mus musculus GN=Hspa1l PE=2 SV=4	0.1	8	0	4	71	12.75	0.001441968
114887.1	sp|P52480|KPYM_MOUSE	Pyruvate kinase PKM OS=Mus musculus GN=Pkm PE=1 SV=4	0.1	8	0	3	58	17.3	0.001441968
105211.1	sp|P10126|EF1A1_MOUSE	Elongation factor 1-alpha 1 OS=Mus musculus GN=Eef1a1 PE=1 SV=3	0.1	7	0	5	50	14.98	0.003026258
105429.1	sp|P17182|ENOA_MOUSE	Alpha-enolase OS=Mus musculus GN=Eno1 PE=1 SV=3	0.1	7	0	2	47	21.23	0.003026258
105507.1	sp|Q8CGC7|SYEP_MOUSE	Bifunctional glutamate/proline--tRNA ligase OS=Mus musculus GN=Eprs PE	0.1	7	0	2	170	3.82	0.003026258
117247.1	sp|O70456|1433S_MOUSE	14-3-3 protein sigma OS=Mus musculus GN=Sfn PE=1 SV=2	7	0.1	2	0	28	12.64	0.003026258
120283.1	sp|P99024|TBB5_MOUSE	Tubulin beta-5 chain OS=Mus musculus GN=Tubb5 PE=1 SV=1	0.1	5	0	3	50	11.08	0.01362796
120124.1	tr|Q9R0T7|Q9R0T7_MOUSE	MCG15085 OS=Mus musculus GN=Try4 PE=2 SV=1	5	0.1	1	0	26	9.52	0.01362796
109186.1	sp|P11499|HS90B_MOUSE	Heat shock protein HSP 90-beta OS=Mus musculus GN=Hsp90ab1 PE=1 SV=	0.1	4	0	4	83	6.01	0.0294083
109258.2	sp|P03975|IGEB_MOUSE	IgE-binding protein OS=Mus musculus GN=Iap PE=2 SV=1	0.1	4	0	2	63	5.58	0.0294083
109719.1	sp|P24547|IMDH2_MOUSE	Inosine-5'-monophosphate dehydrogenase 2 OS=Mus musculus GN=Impdh	0.1	4	0	1	56	6.27	0.0294083
108974.1	sp|P62806|H4_MOUSE	Histone H4 OS=Mus musculus GN=Hist1h4a PE=1 SV=2	4	0.1	2	0	11	17.6	0.0294083
109978.1	sp|Q02257|PLAK_MOUSE	Junction plakoglobin OS=Mus musculus GN=Jup PE=1 SV=3	4	0.1	3	0	82	2.45	0.0294083
101875.1	sp|Q03265|ATPA_MOUSE	ATP synthase subunit alpha, mitochondrial OS=Mus musculus GN=Atp5a1 I	0.1	3	0	2	60	4.19	0.064646504
106570.1	sp|P16858|G3P_MOUSE	Glyceraldehyde-3-phosphate dehydrogenase OS=Mus musculus GN=Gapdh	0.1	3	0	3	36	6.99	0.064646504
110677.1	sp|P06151|LDHA_MOUSE	L-lactate dehydrogenase A chain OS=Mus musculus GN=Ldha PE=1 SV=3	0.1	3	0	2	36	13.71	0.064646504
116659.1	sp|P62908|RS3_MOUSE	40S ribosomal protein S3 OS=Mus musculus GN=Rps3 PE=1 SV=1	0.1	3	0	3	27	11.25	0.064646504
118320.1	sp|Q64337|SQSTM_MOUSE	Sequestosome-1 OS=Mus musculus GN=Sqstm1 PE=1 SV=1	0.1	3	0	2	48	5.19	0.064646504
105969.1	sp|Q14BJ1|FA89A_MOUSE	Protein FAM89A OS=Mus musculus GN=Fam89a PE=2 SV=2	3	0.1	1	0	19	8.03	0.064646504
100183.1	tr|Q9CPN9|Q9CPN9_MOUSE	Protein 2210010C04Rik OS=Mus musculus GN=2210010C04Rik PE=2 SV=1	0.1	2	0	1	26	3.79	0.146611857
104172.1	sp|Q80TT8|CUL9_MOUSE	Cullin-9 OS=Mus musculus GN=Cul9 PE=1 SV=2	0.1	2	0	1	209	0.48	0.146611857
109185.1	sp|P07901|HS90A_MOUSE	Heat shock protein HSP 90-alpha OS=Mus musculus GN=Hsp90aa1 PE=1 SV	0.1	2	0	2	85	2.36	0.146611857
109187.1	sp|P08113|ENPL_MOUSE	Endoplasmin OS=Mus musculus GN=Hsp90b1 PE=1 SV=2	0.1	2	0	1	92	3.79	0.146611857
110468.1	sp|Q3UV17|K22O_MOUSE	Keratin, type II cytoskeletal 2 oral OS=Mus musculus GN=Krt76 PE=2 SV=1	0.1	2	0	1	63	1.59	0.146611857
114886.1	sp|P53657|KPYR_MOUSE	Pyruvate kinase PKLR OS=Mus musculus GN=Pklr PE=2 SV=1	0.1	2	0	1	62	2.41	0.146611857
115629.1	sp|Q9QUM9|PSA6_MOUSE	Proteasome subunit alpha type-6 OS=Mus musculus GN=Psma6 PE=1 SV=1	0.1	2	0	1	27	3.66	0.146611857
115630.1	sp|Q9Z2U0|PSA7_MOUSE	Proteasome subunit alpha type-7 OS=Mus musculus GN=Psma7 PE=1 SV=1	0.1	2	0	2	28	3.59	0.146611857
115643.1	sp|P62192|PRS4_MOUSE	26S protease regulatory subunit 4 OS=Mus musculus GN=Psmc1 PE=1 SV=1	0.1	2	0	1	49	2.03	0.146611857
115644.1	sp|P46471|PRS7_MOUSE	26S protease regulatory subunit 7 OS=Mus musculus GN=Psmc2 PE=1 SV=5	0.1	2	0	1	49	2.06	0.146611857
120282.1	sp|P68372|TBB4B_MOUSE	Tubulin beta-4B chain OS=Mus musculus GN=Tubb4b PE=1 SV=1	0.1	2	0	2	50	5.02	0.146611857
105319.1	sp|P60843|IF4A1_MOUSE	Eukaryotic initiation factor 4A-I OS=Mus musculus GN=Eif4a1 PE=1 SV=1	2	0.1	1	0	46	2.17	0.146611857
110477.1	sp|Q99M73|KRT84_MOUSE	Keratin, type II cuticular Hb4 OS=Mus musculus GN=Krt84 PE=2 SV=2	2	0.1	1	0	65	1.54	0.146611857
114894.1	sp|P97350|PKP1_MOUSE	Plakophilin-1 OS=Mus musculus GN=Pkp1 PE=2 SV=1	2	0.1	1	0	81	1.24	0.146611857
117019.1	sp|Q8K2B3|SDHA_MOUSE	Succinate dehydrogenase [ubiquinone] flavoprotein subunit, mitochondria	4	3	3	2	73	13.1	0.704975995
108023.1	tr|B2RXM2|B2RXM2_MOUSE	EG627828 protein OS=Mus musculus GN=Gm6793 PE=2 SV=1	2	2	1	1	37	5.47	1
120278.1	sp|Q7TMM9|TBB2A_MOUSE	Tubulin beta-2A chain OS=Mus musculus GN=Tubb2a PE=1 SV=1	2	2	2	2	50	7.02	1

Attention: this table may include multiple pages.

NegMG132 vs +MG132- Spectral count comparison of proteins identified from the NegMG132 and +MG132 samples

SC: the summed number of spectral counts (i.e. MS/MS scans) assigned to one identified protein; TP: total number of peptides identified for a protein; Group: Identified proteins that share one or more peptides are grouped together (e.g. 01). The group is represented by the protein with maximal spectra count (e.g. 01.1). If other proteins in the same group are assigned with at least one uniquely identified peptides, these proteins are also counted as unique proteins (e.g. 01.2); Abundance: SC x 50 (KD) / protein size (KD). p value less than 0.05 generally indicates significant change. The p value is derived by G-test (Zhou JY, Afjehi-Sadat L, Asress S, et al. J Proteome Res. 2010, 9:5133-41). The summed SC of the control samples was compared with that of the Test samples.

Group	Reference	Description	SC -MG123	SC +MG123	TP -MG123	TP +MG123	Mass (KD)	Abundance	p-value
108080.1	tr|J3QK04|J3QK04_MOUSE	MCG67952 OS=Mus musculus GN=Gm7808 PE=4 SV=1	0.1	53	0	2	15	180.79	1.98692E-17
107863.1	tr|D3YYZ2|D3YYZ2_MOUSE	MCG1031578 OS=Mus musculus GN=Gm5239 PE=4 SV=1	0.1	51	0	2	14	181.31	8.06923E-17
102095.1	sp|Q9Z1R2|BAG6_MOUSE	Large proline-rich protein BAG6 OS=Mus musculus GN=Bag6 PE=1 SV=1	0.1	21	0	12	121	8.68	1.22518E-07
119792.1	sp|P17751|TPIS_MOUSE	Triosephosphate isomerase OS=Mus musculus GN=Tpi1 PE=1 SV=4	9	0.1	5	0	32	13.99	0.000690662
109198.1	sp|P20029|GRP78_MOUSE	78 kDa glucose-regulated protein OS=Mus musculus GN=Hspa5 PE=1 SV=3	151	109	39	36	72	180.99	0.009042392
102600.1	sp|P35564|CALX_MOUSE	Calnexin OS=Mus musculus GN=Canx PE=1 SV=1	5	0.1	4	0	67	3.72	0.01362796
117019.1	sp|Q8K2B3|SDHA_MOUSE	Succinate dehydrogenase [ubiquinone] flavoprotein subunit, mitochondria	12	3	6	2	73	13.1	0.016187951
109199.1	sp|P63017|HSP7C_MOUSE	Heat shock cognate 71 kDa protein OS=Mus musculus GN=Hspa8 PE=1 SV=	26	44	11	16	71	51.53	0.03049836
100183.1	tr|Q9CPN9|Q9CPN9_MOUSE	Protein 2210010C04Rik OS=Mus musculus GN=2210010C04Rik PE=2 SV=1	0.1	2	0	1	26	3.79	0.146611857
104172.1	sp|Q80TT8|CUL9_MOUSE	Cullin-9 OS=Mus musculus GN=Cul9 PE=1 SV=2	0.1	2	0	1	209	0.48	0.146611857
108023.1	tr|B2RXM2|B2RXM2_MOUSE	EG627828 protein OS=Mus musculus GN=Gm6793 PE=2 SV=1	0.1	2	0	1	37	5.47	0.146611857
110468.1	sp|Q3UV17|K22O_MOUSE	Keratin, type II cytoskeletal 2 oral OS=Mus musculus GN=Krt76 PE=2 SV=1	0.1	2	0	1	63	1.59	0.146611857
114886.1	sp|P53657|KPYR_MOUSE	Pyruvate kinase PKLR OS=Mus musculus GN=Pklr PE=2 SV=1	0.1	2	0	1	62	2.41	0.146611857
115629.1	sp|Q9QUM9|PSA6_MOUSE	Proteasome subunit alpha type-6 OS=Mus musculus GN=Psma6 PE=1 SV=1	0.1	2	0	1	27	3.66	0.146611857
115630.1	sp|Q9Z2U0|PSA7_MOUSE	Proteasome subunit alpha type-7 OS=Mus musculus GN=Psma7 PE=1 SV=1	0.1	2	0	2	28	3.59	0.146611857
115643.1	sp|P62192|PRS4_MOUSE	26S protease regulatory subunit 4 OS=Mus musculus GN=Psmc1 PE=1 SV=1	0.1	2	0	1	49	2.03	0.146611857
115644.1	sp|P46471|PRS7_MOUSE	26S protease regulatory subunit 7 OS=Mus musculus GN=Psmc2 PE=1 SV=5	0.1	2	0	1	49	2.06	0.146611857
100058.1	tr|Q14AA6|Q14AA6_MOUSE	MCG49183 OS=Mus musculus GN=1700009N14Rik PE=2 SV=1	2	0.1	2	0	24	4.11	0.146611857
105555.1	sp|Q91X78|ERLN1_MOUSE	Erlin-1 OS=Mus musculus GN=Erlin1 PE=1 SV=1	2	0.1	1	0	39	2.57	0.146611857
114565.1	sp|Q922R8|PDIA6_MOUSE	Protein disulfide-isomerase A6 OS=Mus musculus GN=Pdia6 PE=1 SV=3	2	0.1	2	0	48	2.08	0.146611857
105429.1	sp|P17182|ENOA_MOUSE	Alpha-enolase OS=Mus musculus GN=Eno1 PE=1 SV=3	13	7	6	2	47	21.23	0.176361396
110677.1	sp|P06151|LDHA_MOUSE	L-lactate dehydrogenase A chain OS=Mus musculus GN=Ldha PE=1 SV=3	7	3	4	2	36	13.71	0.199550991
109187.1	sp|P08113|ENPL_MOUSE	Endoplasmin OS=Mus musculus GN=Hsp90b1 PE=1 SV=2	5	2	4	1	92	3.79	0.249110318
100001.1	cu|NS1_Cus|NS1_Cus|Light	chain Custom sequence	51	41	11	9	27	171.16	0.296669142
114887.1	sp|P52480|KPYM_MOUSE	Pyruvate kinase PKM OS=Mus musculus GN=Pkm PE=1 SV=4	12	8	7	3	58	17.3	0.369477642
109192.1	sp|Q61696|HS71A_MOUSE	Heat shock 70 kDa protein 1A OS=Mus musculus GN=Hspa1a PE=1 SV=2	11	8	4	4	70	13.56	0.490389144
109186.1	sp|P11499|HS90B_MOUSE	Heat shock protein HSP 90-beta OS=Mus musculus GN=Hsp90ab1 PE=1 SV=	6	4	5	4	83	6.01	0.525692869
109194.1	sp|P16627|HS71L_MOUSE	Heat shock 70 kDa protein 1-like OS=Mus musculus GN=Hspa1l PE=2 SV=4	10	8	4	4	71	12.75	0.637004118
101875.1	sp|Q03265|ATPA_MOUSE	ATP synthase subunit alpha, mitochondrial OS=Mus musculus GN=Atp5a1 I	2	3	2	2	60	4.19	0.653629236
106570.1	sp|P16858|G3P_MOUSE	Glyceraldehyde-3-phosphate dehydrogenase OS=Mus musculus GN=Gapdł	2	3	2	3	36	6.99	0.653629236
118320.1	sp|Q64337|SQSTM_MOUSE	Sequestosome-1 OS=Mus musculus GN=Sqstm1 PE=1 SV=1	2	3	2	2	48	5.19	0.653629236
120278.1	sp|Q7TMM9|TBB2A_MOUSE	Tubulin beta-2A chain OS=Mus musculus GN=Tubb2a PE=1 SV=1	3	2	1	2	50	7.02	0.653629236
120282.1	sp|P68372|TBB4B_MOUSE	Tubulin beta-4B chain OS=Mus musculus GN=Tubb4b PE=1 SV=1	3	2	1	2	50	5.02	0.653629236
109258.2	sp|P03975|IGEB_MOUSE	IgE-binding protein OS=Mus musculus GN=Iap PE=2 SV=1	3	4	1	2	63	5.58	0.704975995
109719.1	sp|P24547|IMDH2_MOUSE	Inosine-5'-monophosphate dehydrogenase 2 OS=Mus musculus GN=Impdł	3	4	1	1	56	6.27	0.704975995
109195.1	sp|P17156|HSP72_MOUSE	Heat shock-related 70 kDa protein 2 OS=Mus musculus GN=Hspa2 PE=1 SV	15	17	5	6	70	22.99	0.723587226
105507.1	sp|Q8CGC7|SYEP_MOUSE	Bifunctional glutamate/proline--tRNA ligase OS=Mus musculus GN=Eprs PE	6	7	2	2	170	3.82	0.781406072
105211.1	sp|P10126|EF1A1_MOUSE	Elongation factor 1-alpha 1 OS=Mus musculus GN=Eef1a1 PE=1 SV=3	8	7	5	5	50	14.98	0.796179498
109185.1	sp|P07901|HS90A_MOUSE	Heat shock protein HSP 90-alpha OS=Mus musculus GN=Hsp90aa1 PE=1 SV	2	2	2	2	85	2.36	1
116659.1	sp|P62908|RS3_MOUSE	40S ribosomal protein S3 OS=Mus musculus GN=Rps3 PE=1 SV=1	3	3	2	3	27	11.25	1
120283.1	sp|P99024|TBB5_MOUSE	Tubulin beta-5 chain OS=Mus musculus GN=Tubb5 PE=1 SV=1	5	5	2	3	50	11.08	1
