# Supplementary material for: Identification of two rate-limiting steps in the degradation of partially folded immunoglobulin light chains
Source: Front Cell Dev Biol. 2022 Aug 22;10:924848. doi: 10.3389/fcell.2022.924848 (PMC9441772; doi:10.3389/fcell.2022.924848)
Supplement: Supplementary file 2 [file Presentation1.pdf]

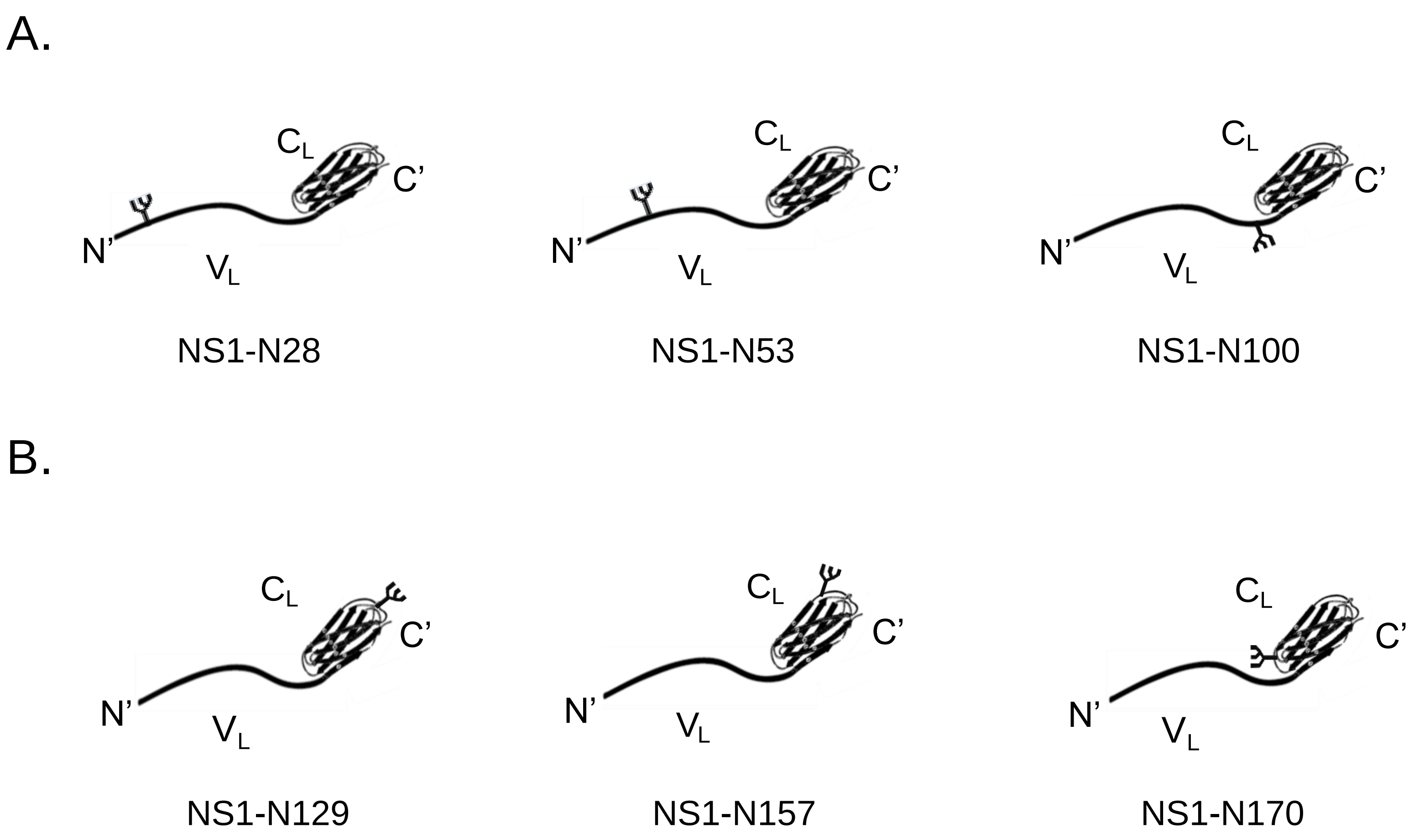

Supplementary Figure S1. Schematic representation to indicate the of location of engineered N-glycans on NS1. Single N-linked glycosylation motifs (S/T-X-N) were engineered throughout the V_L (A) and C_L (B) domains of NS1 at the indicated sites and named for the location of the modified asparagine. The C_L domain is presented as a ribbon structure based on its crystal structure, and glycans were placed on the indicated loops or turns between β strands of this domain.

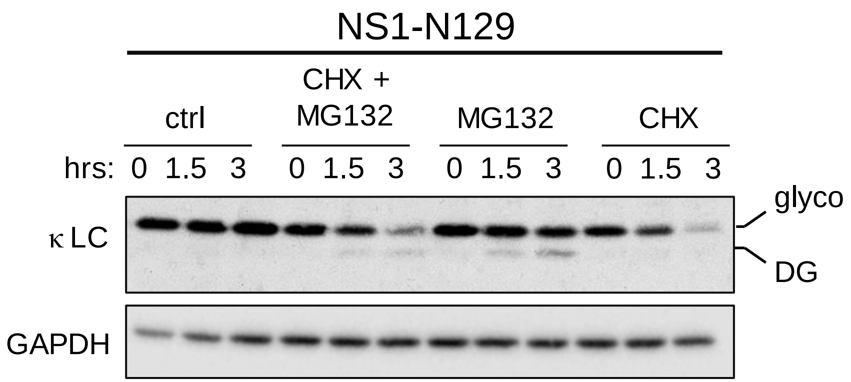

Supplementary Figure S2. Non-glycosylated form of NS1-N129 is not due to the stabilization of a subpopulation that did not enter the ER. Cells expressing the NS1-N129 mutant were incubated with nothing (ctrl), cycloheximide (CHX), MG132 or a combination of cycloheximide and MG132. Lysates were prepared at the indicated time points and analyzed by western blotting as described previously. Deglycosylated (DG) and glycosylated (glyco) species are indicated. GAPDH served as a control for loading.

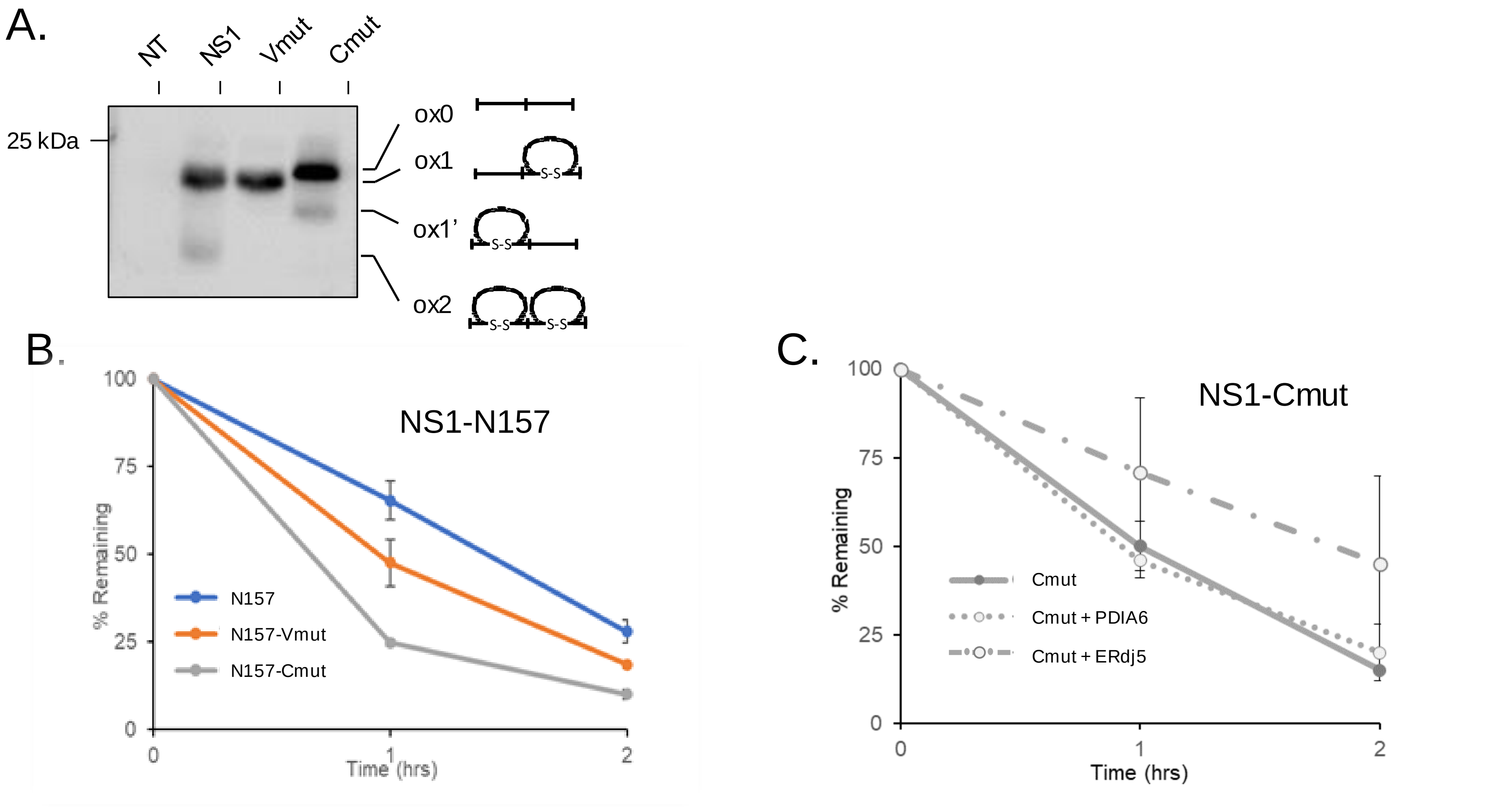

Supplementary Figure S4. Characterization of disulfide-disrupted V_L and C_L domain mutants. (A) Lysates from cells expressing NS1, NS1-V_L domain cysteine mutant (V mut), or NS1-C_L domain cysteine mutant (C mut) were analyzed by non-reducing SDS-PAGE-coupled western blotting with anti-κ LC. The oxidative species are indicated to the right of the gel. (B) Cells expressing the N157-N157 glycosylated version of the disulfide mutants used in Figure 4B were pulse-labeled for 30 min, chased for the indicated times, and κ LC were isolated by immunoprecipitation and signals quantified. (C) Cells were transfected with NS1-Cmut alone or together with either PDIA6 or ERdj5. Pulse-chase experiments were performed as in (B).

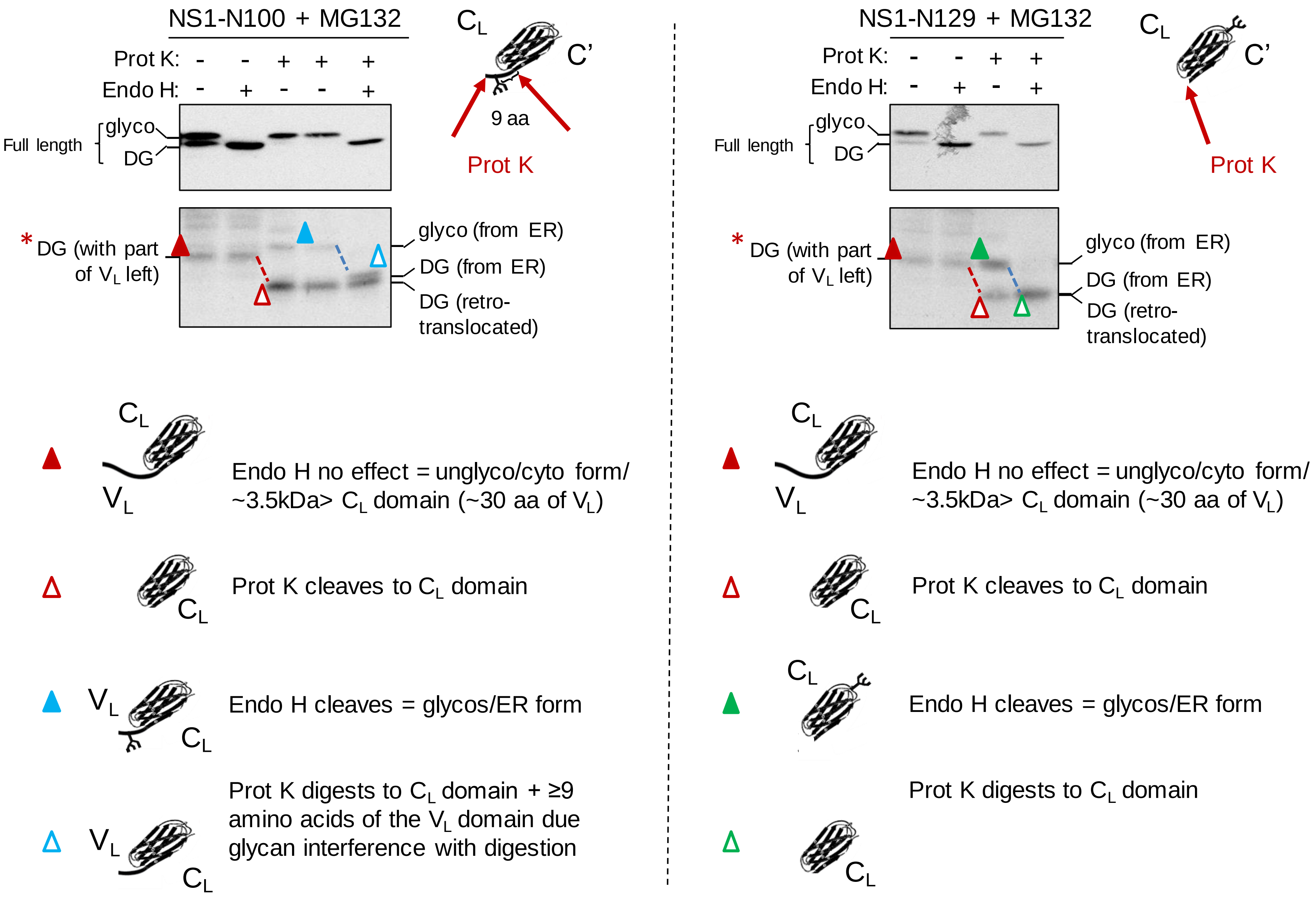

Supplementary Figure S5.

Supplementary Figure S5. Depiction of schematics corresponding to bands indicated in Figure 5. Panel B of Figure 5 was reproduced along with notations on the two panels corresponding to the MG132-treated NS1-N100 and NS1-N129 constructs. Lysates were treated first with Proteinase K and subsequently with Endo H as indicated. The top panels show the full-length proteins and the bottom ones the fragments generated by MG132 treatment alone or combined with proteinase K and Endo H treatment as indicated. The red triangle (▲) denotes an anti-κ reactive band that appears upon MG132 treatment. The mobility of this band is not affected by Endo H treatment indicating that it is not glycosylated and arose from the cytosolic pool that accumulates when the proteasome is inhibited. Proteinase K treatment causes this band to shift downward (red dotted line and Δ), as it was trimmed to the well-folded C_L domain. Based on apparent molecular weight, this reveals that ~30 aa of the V_L is associated with this MG132-induced fragment. A second slower migrating, Proteinase K-generated fragment (▲) was also observed. Endo H treatment increased the mobility of this band (blue dotted line and Δ), indicating that it arose from the ER localized, glycosylated pool of NS1-N100. This species migrated slightly slower than the protease-trimmed C_L domain revealing that the glycan engineered at N100 interfered with cleavage at the V_L:C_L domain boundary, which starts at amino acid 109. The NS1-N129 construct was similarly analyzed. Proteasome inhibition with MG132 generated the same band as identified with the NS1-N100 construct (▲), which was resistant to Endo H cleavage but was trimmed to the C_L domain by Proteinase K digestion (Δ). Protease treatment generated an additional band (▲) that co-migrated with the non-proteinase K digested fragment. Endo H collapsed this band into the C_L domain (Δ) revealing that it had been generated from the ER, glycosylated pool. In this case the position of the glycan does not interfere with proteinase digestion up to the V_L:C_L boundary. Schematics at the bottom portray the composition of the various fragments in cartoon fashion.
